# Sustainability of in vitro light-dependent NADPH generation by the thylakoid membrane of *Synechocystis* sp. PCC6803

**DOI:** 10.1186/s12934-022-01825-1

**Published:** 2022-05-28

**Authors:** Xiaomeng Tong, Eui-Jin Kim, Jeong K. Lee

**Affiliations:** 1 Department of Life Science, Sogang University, Mapo, Shinsu 1, Seoul, 121-742 Korea; 2 Microbial Research Department, Nakdonggang National Institute of Biological Resources, Gyeongsangbuk-do, Sangju-si, 37242 Korea

**Keywords:** Thylakoid membrane, Reducing power, Sustainability, Nox, ROS, Biosilicification

## Abstract

**Background:**

NADPH is used as a reductant in various biosynthetic reactions. Cell-free bio-systems have gained considerable attention owing to their high energy utilization and time efficiency. Efforts have been made to continuously supply reducing power to the reaction mixture in a cyclical manner. The thylakoid membrane (TM) is a promising molecular energy generator, producing NADPH under light. Thus, TM sustainability is of major relevance for its in vitro utilization.

**Results:**

Over 70% of TMs prepared from *Synechocystis* sp. PCC6803 existed in a sealed vesicular structure, with the F_1_ complex of ATP synthase facing outward (right-side-out), producing NADPH and ATP under light. The NADPH generation activity of TM increased approximately two-fold with the addition of carbonyl cyanide-p-(trifluoromethoxy) phenylhydrazone (FCCP) or removal of the F_1_ complex using EDTA. Thus, the uncoupling of proton translocation from the electron transport chain or proton leakage through the F_o_ complex resulted in greater NADPH generation. Biosilicified TM retained more than 80% of its NADPH generation activity after a week at 30°C in the dark. However, activity declined sharply to below 30% after two days in light. The introduction of engineered water-forming NADPH oxidase (Nox^m^) to keep the electron transport chain of TM working resulted in the improved sustainability of NADPH generation activity in a ratio (Nox^m^ to TM)-dependent manner, which correlated with the decrease of singlet oxygen generation. Removal of reactive oxygen species (ROS) by catalase further highlighted the sustainable NADPH generation activity of up to 80% in two days under light.

**Conclusion:**

Reducing power generated by light energy has to be consumed for TM sustainability. Otherwise, TM can generate singlet oxygen, causing oxidative damage. Thus, TMs should be kept in the dark when not in use. Although NADPH generation activity by TM can be extended via silica encapsulation, further removal of hydrogen peroxide results in an improvement of TM sustainability. Therefore, as long as ROS formation by TM in light is properly handled, it can be used as a promising source of reducing power for in vitro biochemical reactions.

**Graphical Abstract:**

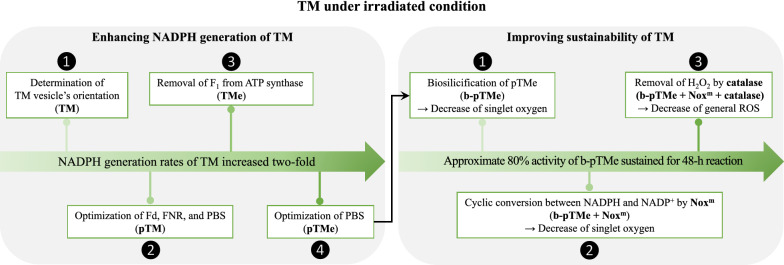

**Supplementary Information:**

The online version contains supplementary material available at 10.1186/s12934-022-01825-1.

## Introduction

The thylakoid of cyanobacteria houses photosynthetic machinery. This membranous structure is composed of flattened sacs that are organized into stacks [1, 2]. Further, it is the site of light-dependent photosynthesis, harboring components required for electron transport involved in both linear electron flow (LEF) and cyclic electron flow (CEF) [3, 4]. LEF is utilized for the generation of NADPH and ATP, whereas CEF is exclusively involved in ATP production [5, 6].

The main module for light-driven electron flow comprises the water-splitting complex, photosystem II (PS II), cytochrome *b*_*6*_*f* (cyt *b*_*6*_*f*), photosystem I (PS I), and electron carriers of plastoquinone (PQ) in the membrane, in addition to plastocyanin (PC) in the lumen [3, 4, 7, 8]. The phycobilisome (PBS) acts as a light-harvesting complex and transfers light energy to PS II [9]. Ferredoxin (Fd) is finally reduced and used as a substrate for ferredoxin-NADP^+^ oxidoreductase (FNR) to form NADPH [10]. Meanwhile, the proton motive force (pmf) is formed across the TM, thus driving ATP production by ATP synthase.

The vesicular structure of the TM can be readily formed following isolation from phototrophic organisms. The TM from *Spinacia oleracea* was shown to generate NADPH and ATP under light and was used for CO_2_ fixation and poly (3-hydroxybutyrate) production in vitro [11, 12]. TM from *Chlamydomonas reinhardtii* has also been used to study H_2_ generation in vitro [13]. Thus, the TM represents a promising light-dependent energy generator for biochemical reactions in vitro. However, the limited in vitro durability of TMs limits their long-term utility [11, 12].

The sustainability of chromatophore membrane vesicles derived from the intracytoplasmic membrane of *Rhodobacter sphaeroides* has been improved by immobilization on streptavidin resin [14]. However, the use of resins in large quantities is not cost-effective. Recently, biosilicification has attracted a lot of attention, as some bacteria naturally form a silica shell that protects against environmental stress [15–17]. In this process, silicic acid is converted into polymerized silica under physiological conditions [18]. Over the past few decades, microorganism-coating biomimetic silica has been utilized on account of numerous merits. Silica coating not only preserves cell viability, but also offers a rigid protective layer against external stress [19–21]. The porous nature of silica-based shields enable cells to uptake necessary metabolites from the culture medium [22, 23]. Moreover, silica-mediated immobilization of enzymes facilitates their reusability without compromising stability [24]. Encapsulated proteins confined by silica shells are known to possess considerable resistance to acidic environment, digestive enzymes, and denaturing agents [25–27]. However, if the thickness of silica shell exceeds a certain limit, the movement of reactants across silica is restricted and would slow down the reaction [26].

The long-term exposure of TM to light may impair its biocatalyst activity, which could be ascribed to the unstable properties of the lipid bilayer [28] and reactive oxygen species (ROS)-induced oxidative damage to membrane lipids and proteins [29]. The redox poise of electron carriers must be maintained throughout electron transport during photosynthesis. Otherwise, a reduced carrier can liberate electrons to molecular oxygen to generate ROS [30, 31].

In this study, we sought ways to improve the sustainability of NADPH generation by TMs, since there are various other methods of producing ATP in vitro [32, 33]. Considering surface protection, biomimetic silicification was used to wrap up the TM and test whether it improves sustainability. Furthermore, ROS formation by the TM was examined during active light exposure. To provide cyclic conversion between NADPH and NADP^+^, an engineered water-forming NADPH oxidase (Nox^m^) was applied after introducing mutations to gain substrate preference for NADPH over NADH [34].

## Materials and methods

### Bacterial strains and growth conditions

*Synechocystis* sp. PCC6803 (hereafter referred to as *Synechocystis*) was grown at 30°C in BG-11 medium with 10 mM glucose, as previously described [35]. The culture broth was agitated at 110 rpm under 50 μmol m^−2^ s^−1^ white light. Cells for TM isolation were harvested at an OD_730_ in the range of 1.8–2.5. *Escherichia coli* was cultivated in Luria–Bertani (LB) broth [36]. Antibiotics were supplied as described hereafter when necessary [36]. Kanamycin (Km) and ampicillin (Ap) were added at 25 µg mL^−1^ and 50 µg mL^−1^ for *E. coli*, respectively. Gentamicin (Gm) was used at 30 µg mL^−1^ for both *Synechocystis* and *E. coli*. To induce protein expression, 1 mM IPTG was added to both strains. Anhydrotetracycline was used at 200 ng mL^−1^ for *E. coli*.

### Plasmid construction

The plasmids used to determine the orientation of TMs were constructed by introducing a his_6_-tag into the N-terminus of the β subunit and a strep-tag into the N-terminus of the c subunit of ATP synthase via PCR with primers harboring tag sequences. Briefly, a 1.5-kb fragment containing the β subunit (*atpB*) was PCR-amplified from *Synechocystis* genomic DNA using primers AtpB-F1 and AtpB-R1 (Additional file 1: Table S1). The resulting fragment was digested with *Bam*HI and *Eco*RI, followed by ligation into the *Bam*HI/*Eco*RI sites of pSL1211 [37] to generate pSL-AtpB (Table 1), which was expressed under the control of the IPTG-inducible *trc* promoter. A 246-bp DNA fragment containing the c subunit (*atpH*) was PCR amplified using primers AtpH-F and AtpH-R (Additional file 1: Table S1), digested with *Bam*HI and *Eco*RI, and ligated into the *Bam*HI/*Eco*RI sites of pSL1211 to yield pSL-AtpH (Table 1).

**Table 1:** Strains and plasmids used in this study

Strains/plasmids	Relevant characteristics	Reference/description
Strains		
*E. coli*		
DH5α*phe*	*phe*::Tn10*d*Cm of DH5α	[74]
S17-1	C600::RP4 2-(Tc::Mu)(Km::Tn7) *thi pro hsdR hsdM*^+^ *recA*	[75]
BL21(DE3)	B F- *dcm ompT hsdS*(r_B_^−^m_B_^−^) *gal* λ(ED3)	
*Synechocystis sp. PCC 6803*	Type strain	
Syn-AtpB	*Synechocystis* containing pSL-AtpB	This study
Syn-AtpH	*Synechocystis* containing pSL-AtpH	This study
Plasmids		
pSL1211	*ori* RSF1010, IPTG-inducible promoter; Gm^r^	[37]
pSL-AtpB	pSL1211 + 1.5-kb *Bam*HI-*Eco*RI fragment containing *atpB*; Gm^r^	This study
pSL-AtpH	pSL1211 + 246-bp *Bam*HI-*Eco*RI fragment containing *atpH*; Gm^r^	This study
pASK-IBA3plus	*ori* f1, Ap^r^; For expression of recombinant protein with C-terminal strep-tag	IBA Lifesciences
IBA-AtpA	pASK-IBA3plus + 1.5-kb *Bsa*I fragment containing *atpA*; Ap^r^	This study
IBA-AtpB	pASK-IBA3plus + 1.5-kb *Bsa*I fragment containing *atpB*; Ap^r^	This study
IBA-AtpD	pASK-IBA3plus + 558-bp *Bsa*I fragment containing *atpD*; Ap^r^	This study
IBA-AtpE	pASK-IBA3plus + 498-bp *Bsa*I fragment containing *atpE*; Ap^r^	This study
IBA-KatE	pASK-IBA3plus + 1.5-kb *Bam*HI-*Pst*I fragment containing *katE*; Ap^r^	This study
pASK-IBA7plus	*ori* f1, Ap^r^; For expression of recombinant protein with N-terminal strep-tag	IBA Lifesciences
IBA-FNR	pASK-IBA7plus + 1.2-kb *Bam*HI-*Pst*I fragment containing *petH*; Ap^r^	This study
IBA-Fd	pASK-IBA7plus + 369-bp *Eco*RI-*Pst*I fragment containing *petF*; Ap^r^	This study
IBA-ApcA	pASK-IBA7plus + 486-bp *Bam*HI-*Pst*I fragment containing *apcA*; Ap^r^	This study
IBA-CpcA	pASK-IBA7plus + 489-bp *Bam*HI-*Pst*I fragment containing *cpcA*; Ap^r^	This study
pGEX-4T-3	pBR322 *ori*, Ap^r^; For expression of recombinant protein with N-terminal GST-tag	This study
pGEX-AtpC	pGEX-4T-3 + 498-bp *Eco*RI-*Xho*I fragment containing *atpC*; Ap^r^	This study
pRSET-A	pBR322 *ori*, Ap^r^; For expression of recombinant protein with N-terminal his_6_-tag	Invitrogen
pRSET-Nox	pRSET-A + 1.3-kb *Bam*HI-*Pst*I fragment containing *noxV*; Ap^r^	This study
pRSET-Nox178	pRSET-A + 1.3-kb *Bam*HI-*Pst*I fragment containing *noxG178R*; Ap^r^	This study
pRSET-Nox178179	pRSET-A + 1.3-kb *Bam*HI-*Pst*I fragment containing *noxG178RL179R*; Ap^r^	This study

A 1.5-kb DNA fragment containing ATP synthase subunit α (*atpA*) was PCR-amplified from *Synechocystis* genomic DNA using the primers AtpA-F and AtpA-R (Additional file 1: Table S1). The PCR product was digested with *Bsa*I and ligated into the *Bsa*I site of pASK-IBA3plus with a C-terminal strep-tag fusion to yield IBA-AtpA (Table 1).

A 1.5-kb DNA fragment containing ATP synthase subunit β (*atpB*) was PCR-amplified from *Synechocystis* genomic DNA using primers AtpB-F2 and AtpB-R2 (Additional file 1: Table S1). The PCR product was digested with *Bsa*I and ligated into the *Bsa*I site of pASK-IBA3plus with a C-terminal strep-tag fusion to yield IBA-AtpB (Table 1).

A 432-bp DNA fragment containing ATP synthase subunits γ (*atpC*) was PCR-amplified from *Synechocystis* genomic DNA using primers AtpC-F and AtpC-R (Additional file 1: Table S1). The resulting fragment was digested with *Eco*RI and *Xho*I, followed by ligation into the *Eco*RI/*Xho*I sites of pGEX-4T-3 with N-terminal GST-tag fusion to yield pGEX-AtpC (Table 1).

A 558-bp DNA fragment containing ATP synthase subunits δ (*atpD*) was PCR-amplified from *Synechocystis* genomic DNA using the primers AtpD-F and AtpD-R (Additional file 1: Table S1). The PCR product was digested with *Bsa*I and ligated into the *Bsa*I site of pASK-IBA3plus with a C-terminal strep-tag fusion to yield IBA-AtpD (Table 1).

A 498-bp DNA fragment containing ATP synthase subunit ε (*atpE*) was PCR-amplified from *Synechocystis* genomic DNA using primers AtpE-F and AtpE-R (Additional file 1: Table S1). The PCR product was digested with *Bsa*I and ligated into the *Bsa*I site of pASK-IBA3plus with a C-terminal strep-tag fusion to yield IBA-AtpE (Table 1).

A 1.2-kb fragment containing FNR (*petH*) was PCR-amplified from *Synechocystis* genomic DNA using primers FNR-F and FNR-R (Additional file 1: Table S1). The PCR product was digested with *Bam*HI and *Pst*I, and ligated into the *Bam*HI/*Pst*I sites of pASK-IBA7plus to generate IBA-FNR (Table 1) with an N-terminal strep-tag fusion.

A 369-bp fragment containing Fd (*petF*) was PCR-amplified from *Synechocystis* genomic DNA using primers Fd-F and Fd-R (Additional file 1: Table S1). The PCR product was digested with *Eco*RI and *Pst*I, and ligated into the *Eco*RI/*Pst*I sites of pASK-IBA7plus to generate IBA-Fd (Table 1) with an N-terminal strep-tag fusion.

A 486-bp fragment containing the allophycocyanin α subunit (*apcA*) was PCR-amplified from *Synechocystis* genomic DNA using primers ApcA-F and ApcA-R (Additional file 1: Table S1). The PCR product was digested with *Bam*HI and *Pst*I, and ligated into the *Bam*HI/*Pst*I sites of pASK-IBA7plus to generate IBA-ApcA (Table 1) with an N-terminal strep-tag fusion.

A 489-bp fragment containing the phycocyanin α subunit (*cpcA*) was PCR-amplified from *Synechocystis* genomic DNA using primers CpcA-F and CpcA-R (Additional file 1: Table S1). The PCR product was digested with *Bam*HI and *Pst*I, and ligated into the *Bam*HI/*Pst*I sites of pASK-IBA7plus to generate IBA-CpcA (Table 1) with an N-terminal strep-tag fusion.

A 1.5-kb fragment containing water-forming NADH oxidase (Nox) (*noxV*) was PCR-amplified from *Lactobacillus plantarum* genomic DNA with the primers Nox-F/R (Additional file 1: Table S1). The PCR product was digested with *Bam*HI and *Pst*I, and ligated into the *Bam*HI/*Pst*I sites of pRSET-A to produce pRSET-Nox (Table 1) with an N-terminal his_6_-tag fusion. The mutant enzyme NoxG178R was generated from pRSET-Nox with the primers Nox178-F and Nox178-R (Additional file 1: Table S1) using PrimeSTAR Max (Takara Bio, Japan). The resulting PCR product was treated with *Dpn*I to destroy the methylated parental DNA, followed by transformation into *E. coli*. Plasmid pRSET-Nox178 (Table 1), harboring the G178R mutation, was validated through sequencing. Accordingly, primers Nox178179-F and Nox178179-R (Additional file 1: Table S1) were used to construct pRSET-Nox178179 (Table 1) from pRSET-Nox178, as described above. It has G178R and L179R double-mutated sites, which were verified via sequencing.

A 1.5-kb fragment containing catalase (*katE*) was PCR-amplified from *Vibrio vulnificus* genomic DNA using primers KatE-F and KatE-R (Additional file 1: Table S1). The PCR product was digested with *Bam*HI and *Pst*I, and ligated into the *Bam*HI/*Pst*I sites of pASK-IBA3plus to generate IBA-KatE (Table 1) with a C-terminal strep-tag fusion.

### Plasmid conjugation

Plasmids were mobilized from *E. coli* S17-1 to *Synechocystis* as previously described [35]. Exponential phase *E. coli* S17-1 harboring pSL-AtpB or pSL-AtpH and *Synechocystis* were mixed in a 1:1 ratio (v/v). The mating mixture was left on BG-11 medium containing 5% LB at 30°C for 12 h under white light (50 μmol m^−2^ s^−1^). The exconjugants carrying pSL-AtpB or pSL-AtpH were selected on BG-11 medium containing Gm.

### Purification of TM from *Synechocystis*

TMs were purified as previously described [7], with some modifications*.* Cells were harvested from 1 L of culture and suspended in TM buffer (10 mM sodium phosphate (pH 7.5), 5% sucrose, 10 mM MgCl_2_, 5 mM sodium ascorbate, and 5% betaine) on ice, followed by disruption by sonication for 5 min for a total of three times. Ascorbate was added to the buffer to quench singlet oxygen. Cell debris was removed by centrifugation at 5000*g* for 10 min at 4°C. The resulting supernatant was subjected to ultracentrifugation at 150,000*g* and 4°C for 1 h to obtain the total cell membrane. It was suspended in 2 mL ice-cold TM buffer and loaded onto a discontinuous sucrose density gradient (60%, 40%, 20% (w/v)), followed by ultracentrifugation at 150,000*g* and 4°C for 3 h. The chlorophyll a (Chl *a*)-condensed layer between 40 and 20% sucrose was collected as the TM fraction, which was then diluted with an equal volume of 10 mM sodium phosphate buffer (pH 7.5) and kept at −80°C in the dark for further analysis.

### Determination of Chl *a* content

The Chl *a* content of the TM was determined as previously described [38]. Briefly, 10 µL of purified TM was dissolved in 500 μL acetone/methanol (7/2, v/v) via a brief vortex. This was followed by centrifugation at 15,000*g* at 4°C for 1 min. The pigment content was determined with an extinction coefficient of ε_660_ = 77.1 mM^−1^ cm^−1^ [38].

### Preparation of TMs harboring his_6_-tagged β (TM_β-his_) and strep-tagged c (TM_c-strep_) subunits of ATP synthase

We determined whether ATP synthase enzymes of the TM vesicles are oriented outward or inward through previously described methodology [39], with some modifications. The his_6_-tagged β subunit and strep-tagged c subunit were assembled into ATP synthase in the recombinant strains Syn-AtpB and Syn-AtpH (Table 1), respectively. TM_β-his_ and TM_c-strep_ were prepared and loaded onto columns containing Ni–NTA and streptavidin resins within a linear range of binding capacity. The flow-through fraction (FF) was interpreted to have TM vesicles containing the inside orientation of the tag, whereas the elution fractions (EF) were interpreted to have an outside orientation of the tag. The FF and EF were adjusted to the same volume before separation via SDS-PAGE. The wash fraction (WF) was included as a control. Proteins were transferred to polyvinylidene difluoride (PVDF) membranes and probed with anti-his_6_-tag antibody (D291-3, MBL) or anti-strep-tag antibody (2-1507-001, IBA Life Sciences). An HRP-conjugated anti-mouse antibody (#7076, Cell Signaling Technology) was used to visualize the signal, which was detected by reaction with an ECL working solution (iNtRON Biotechnology, Korea).

To determine the tagging % from the total contents of subunits β and c, the TMs were pretreated with 0.5% Triton X-100 prior to affinity chromatography. The following procedure was performed as described above. One of the duplicated protein blots was further examined with either an anti-β antibody (generated using immunogen of β subunit, see below) or anti-c antibody (AS05 071, Agrisera) to determine the extent to which the tagged subunit was incorporated into the ATP synthase of the TM. HRP-conjugated anti-rat antibody (sc-2006, Santa Cruz Biotechnology) and anti-rabbit antibody (#7074, Cell Signaling Technology) were used to probe the β- and c-subunits, respectively. The bands were visualized by reaction with ECL working solution, and all quantifications were processed using ImageJ.

### Purification of PBS from *Synechocystis*

PBS was isolated from *Synechocystis* as previously described [40]. Cells were harvested and suspended in 0.8 M potassium phosphate buffer (pH 7.0) before breakage by sonication. Supernatant from the cell lysate was treated with 2% Triton X-100 at 20°C for 30 min and subjected to ultracentrifugation at 150,000*g* and 20°C for 20 min. The clear blue layer containing PBS was collected and loaded onto a sucrose density gradient (1 mL of 2.0 M, 3 mL of 1.0 M, 2.5 mL of 0.75 M, 2.5 mL of 0.5 M, 2 mL of 0.25 M sucrose in 0.8 M potassium phosphate buffer (pH 7.0)), followed by ultracentrifugation at 150,000*g* and 20℃ for 16 h. PBS was obtained at the layer between 1.0 M and 0.75 M sucrose. It was then stored at 4°C in the dark until further use. Phycocyanin and allophycocyanin concentrations were calculated using the following equations: [41]

$${\text{Phycocyanin}}{\mkern 1mu} = {\mkern 1mu} \left( {{\text{A}}_{{615}} - 0.474*{\text{A}}_{{652}} } \right)/5.34\,\left( {{\text{mg mL}}^{{ - 1}} } \right)$$

$${\text{Allophycocyanin}}\, = \,\left( {{\text{A}}_{{{652}}} - 0.{2}0{8}*{\text{A}}_{{{615}}} } \right)/{5}.0{9 }\,\left( {{\text{mg mL}}^{{ - {1}}} } \right)$$

Purified PBS consisted of phycocyanin and allophycocyanin at a ratio of approximately 2:1 (w/w).

### EDTA treatment of TM

The F_1_ complex of ATP synthase was removed from the TM with EDTA, as previously described [42, 43], with some modifications. TM containing 20 μg Chl *a* mL^−1^ was mixed with varying concentrations of EDTA in Mg-free TM buffer (10 mM sodium phosphate (pH 7.5), 5% sucrose, 5 mM sodium ascorbate, and 5% betaine) and incubated at 4°C for 1 h. The mixture was then subjected to sonication on ice, six times for 30 s each (with a 50% duty cycle). The EDTA-treated TM (TMe) was washed twice with TM buffer by ultracentrifugation at 150,000*g* and 4°C for 1 h. TMe was kept at 4°C in the dark until use.

### Reconstitution of TM with PBS

TM (or TMe) containing 5 μg Chl *a* mL^−1^ was mixed with varying levels of PBS (One equivalent of PBS consists of phycocyanin (3.6 µg mL^−1^) and allophycocyanin (1.8 µg mL^−1^)) in a total volume (4 mL) of TM buffer, followed by incubation at room temperature for 20 min in dark. Free PBS was removed by ultracentrifugation at 150,000*g* and 4°C for 1 h. The resulting PBS-reconstituted TM (pTM) (or PBS-reconstituted TMe (pTMe)) was kept at 4°C in the dark until use.

### Determination of ATP and NADPH generation activity of TMs

ATP and NADPH generation activities were measured using TM (or pTM) at 5 μg Chl *a* mL^−1^ in TM buffer supplemented with 1 μM FNR, 10 μM Fd, 2 mM NADP^+^, and 2 mM ADP (TM-NADP^+^-ADP buffer). When TM was deprived of the F_1_ complex of ATP synthase with EDTA, NADPH generation activity was determined using TM buffer supplemented with 1 μM FNR, 10 μM Fd, and 2 mM NADP^+^ (TM-NADP^+^ buffer). The reaction mixture was incubated at 30°C under white light (50 μmol m^−2^ s^−1^). The reaction in the dark was used as a control. Aliquots of samples were taken at 30-min intervals and stored at −80°C prior to analysis. NADPH was detected based on changes in the absorbance at 340 nm over a time-course and calculated with an extinction coefficient of 6.22 mM^−1^ cm^−1^, whereas ATP was determined using an ATP Colorimetric/Fluorometric Assay Kit (K354, BioVision). All quantifications were independently repeated three times, and data are shown as the mean ± standard deviation (SD).

### Treatment of pTM with carbonyl cyanide-p-(trifluoromethoxy) phenylhydrazone (FCCP)

pTM containing 5 μg Chl *a* mL^−1^ was added to TM-NADP^+^-ADP buffer supplemented with varying levels (0–10 μM) of FCCP. The mixtures were incubated at room temperature for 20 min in the dark, followed by the measurement of ATP and NADPH generation, as described above.

### Preparation or acquisition of antiserums for western blot analysis

Each subunit (α, β, γ, δ, and ε) of ATP synthase as well as each α subunit of allophycocyanin and phycocyanin in PBS was expressed in *E. coli* BL21(DE3) and purified for use as immunogens to prepare rat antiserum as previously described [44]. The resulting antisera were used to probe the target bands at a dilution of 1:2000 for western blot analysis, as previously described [45].

Antibodies PsbO (AS06 142–33), PsbA (AS10 703), PC (AS06 141), and PsaC (AS10 939) were purchased from Agrisera (Sweden) and used according to the manufacturer’s instructions in order to examine the stability of TM photosynthetic machinery. The target bands that reacted with the above antibodies were incubated with an HRP-conjugated anti-rat antibody (sc-2006, Santa Cruz Biotechnology) or an anti-rabbit antibody (#7074, Cell Signaling Technology) to develop signals using ECL working solution, followed by quantification with ImageJ.

### Biosilicification of pTMe

pTMe was biosilicified as previously described [46]. pTMe containing 10 µg Chl *a* mL^−1^ was mixed with R5 (synthesized by Anygen, Korea) at 0.5 mg mL^−1^ in TM buffer and incubated at room temperature for 5 min. Silicic acid was prepared as previously described [46]. Briefly, tetramethyl orthosilicate (TMOS) at 1 M was prehydrolyzed at room temperature for 30 min in 1 mM hydrochloric acid. TMOS was added at a concentration of 10 mM to pTMe pretreated with R5, and biosilicification proceeded by incubating the mixture at room temperature for 30 min. The resulting biosilicified pTMe (b-pTMe) was washed three times with TM buffer via centrifugation at 3000*g* at 4°C. The resulting pellet was suspended in TM buffer and stored at 4°C in the dark until further use.

### Examination of zeta potential

pTMe, pTMe pre-incubated with R5 as described above, and b-pTMe were prepared at 5 µg Chl *a* mL^−1^ for the measurement of zeta potential with a Zetasizer Nano S (Malvern, UK). pTMe pre-incubated with R5 was washed twice with TM buffer via ultracentrifugation at 150,000*g* and 4°C for 30 min to remove any free R5. All quantifications were independently repeated three times, and data are shown as the mean ± standard deviation (SD).

### Determination of pTMe and b-pTMe sustainability

pTMe and b-pTMe were prepared at 5 µg Chl *a* mL^−1^ in 10 mL of TM-NADP^+^ buffer (10 mL). Reaction mixtures were incubated at 30°C in the dark for one week or in white light (50 μmol m^−2^ s^−1^) for two days. Aliquots (0.5 mL) were withdrawn intermittently from each sample and washed with TM buffer three times via ultracentrifugation at 150,000*g* and 4°C for 30 min prior to the activity assays. NADPH production was determined in TM-NADP^+^ buffer at 30°C under white light (50 μmol m^−2^ s^−1^).

### Examination of R5 antioxidant activity

Singlet oxygen generation and quenching were examined as previously described [47]. To determine the antioxidant activity of R5 peptide, varying levels of R5 were prepared in 0.1 mM phosphate buffer (pH 7.0), followed by the addition of linoleic acid in ethanol to a final concentration of 50 mM. Histidine (3 mM) was used as a positive control. The reaction mixtures were placed under white light (50 μmol m^−2^ s^−1^) after the addition of 0.1 mM methylene blue. Samples were withdrawn at 1-h intervals. The peroxide content was measured via the ferric thiocyanate method [48]. Ferric thiocyanate complex was detected at 500 nm. Quenching of singlet oxygen was calculated as inhibition % using the following equation: Inhibition (%) = (A_*0*_–A_*1*_)/*A*_*0*_ × 100, where *A*_*0*_ is the absorption of the control after 1-h irradiation, and *A*_*1*_ is the absorption of samples containing R5 or histidine after the same period of irradiation.

The superoxide scavenging activity of R5 was measured using xanthine and xanthine oxidase (Roche) in the presence of nitroblue tetrazolium chloride (NBT) [49]. Xanthine is oxidized by xanthine oxidase to generate superoxide, which can oxidize NBT to formazan, as indicated by the absorbance at 560 nm. The reaction mixture (1 mL) consisted of varying concentrations of R5, 0.2 mM xanthine, 0.5 U mL^−1^ xanthine oxidase, and 0.5 mM NBT. Bovine superoxide dismutase (SOD) (5 U mL^−1^) (Sigma S9697) was used as the positive control. The reaction was performed at room temperature for 20 min, and the scavenging activity of superoxide was calculated as inhibition % using the following equation: Inhibition (%) = (A_*0*_–A_*1*_)/*A*_*0*_ × 100, where *A*_*0*_ is the absorption of the control after a 20-min reaction, and *A*_*1*_ is the absorption of samples containing R5 or bovine SOD after reaction for the same period.

Hydrogen peroxide (H_2_O_2_) scavenging assays were performed as described previously [50]. The reaction mixture (0.5 mL) consisted of varying concentrations of R5 and 100 mM H_2_O_2_ in 10 mM phosphate buffer (pH 7.5). Catalase (30 µg mL^−1^) was used as the positive control. After reaction at 30°C for 5 min, the absorption at 230 nm was monitored. The scavenging activity of hydrogen peroxide was calculated as inhibition % using the following equation: Inhibition (%) = (A_*0*_–A_*1*_)/*A*_*0*_ × 100, where *A*_*0*_ is the absorption of the samples at time zero, and *A*_*1*_ is the absorption of the sample after reaction for 5 min.

### Purification of water-forming NADPH oxidase and determination of its activity

Water-forming NADPH oxidase (Nox^m^) was produced from *E. coli* BL21(DE3) harboring pRSET-Nox178179 (Table 1) and purified using Ni–NTA resin as previously described [51]. The NADPH oxidation activity of Nox^m^ was determined as previously described [34]. The reaction mixture (0.5 mL) consisted of varying concentrations of NADPH (0–100 µM) in 10 mM phosphate buffer (pH 7.5). The assay was conducted at 30°C, and NADPH was determined at 340 nm with an extinction coefficient of 6.22 mM^−1^ cm^−1^.

### Detection of ROS generated by pTMe and b-pTMe

The singlet oxygen generated by pTMe and b-pTMe in light was determined using Singlet Oxygen Sensor Green (SOSG) (Invitrogen™, S36002), as previously described [52, 53]. SOSG was prepared at 50 µM in TM-NADP^+^ buffer (0.2 mL) containing either pTMe or b-pTMe (5 µg Chl *a* mL^−1^). The mixture was added into a black 96-well microtiter plate covered with a transparent lid. After incubation at 30°C for 30 min under 50 μmol m^−2^ s^−1^ white light, fluorescence was detected at 525 nm with excitation at 504 nm. Measurements were performed within a linear relationship between the levels of pTMe (or b-pTMe) and the fluorescence signal at 525 nm. Fluorescence from SOSG without pTMe or b-pTMe addition was used as a negative control. All quantifications were independently repeated three times, and data are shown as the mean ± standard deviation (SD).

General ROS generated by pTMe and b-pTMe in light, including hydrogen peroxide, hydroxyl radicals, peroxyl radicals, and peroxynitrite, were detected with H_2_DCFDA (Sigma-Aldrich). Either pTMe or b-pTMe (each at 5 µg Chl *a* mL^−1^) was mixed with TM-NADP^+^ buffer and incubated at 30°C for 2 h under 50 μmol m^−2^ s^−1^ white light. Aliquots (0.2 mL) were withdrawn and lysed with 4 µL chloroform, followed by vigorous vortexing. The samples were transferred to a black 96-well microtiter plate containing H_2_DCFDA (0.1 mM) and the cell lysate (32 µg) of *Synechocystis* as an esterase source to detect general ROS after incubation at 30°C in the dark for 10 min. Fluorescence at 525 nm was detected within a linear relationship with the level of pTMe (or b-pTMe) after excitation at 488 nm. The reaction without pTMe or b-pTMe was used as a negative control. All quantifications were independently repeated three times, and data are shown as the mean ± standard deviation (SD).

## Results and discussion

### Examination of structure of the purified TM

Two different types of vesicles can be obtained when TMs are prepared via sonication cell lysis: ATP synthase F_1_-out and F_1_-in orientation [54, 55]. An F_1_-out vesicle is the structure that can generate NADPH and ATP using light. To evaluate the proportion of F_1_-out vesicles, the β (F_1_)-and c (F_o_)-subunits of ATP synthase were analyzed for membrane orientation. Previously, the his_6_-tagged β-subunit was introduced into the ATP synthase of artificial proteoliposomes, and the membrane orientation was determined by inspecting its binding to Ni–NTA magnetic beads [39].

The recombinant β-subunit with a his_6_-tag at its N-terminus was produced in Syn-AtpB (Table 1), and TM was obtained from cells via sucrose density gradient ultracentrifugation. In order to detect F_1_-out vesicles, varying amounts of TM, which were determined with Chl *a* unless stated otherwise, were loaded onto the Ni–NTA column within the binding capacity of the resin for the tag, as previously described [14]. Unbound free TM was removed by washing, and the Chl *a* content of resin-bound TM_β-his_ was determined after acetone/methanol extraction. The maximum binding capacity was approximately 3.4 µg Chl *a* per mL Ni–NTA resin (Additional file 1: Fig. S1A).

**Fig. 1:**
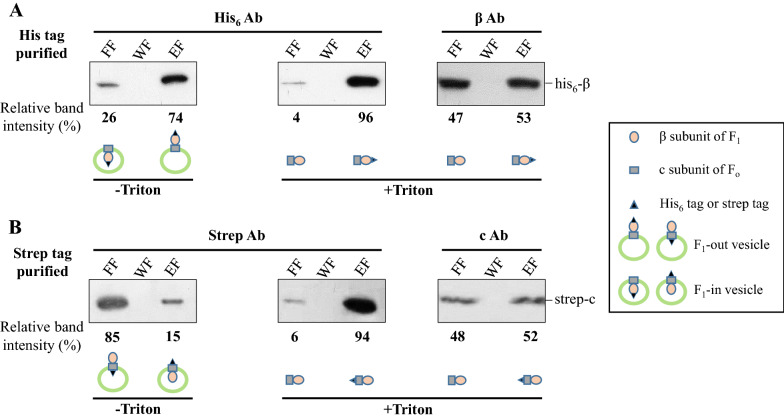
Structure of the purified TM. **A** TM prepared from Syn-AtpB contains the his_6_-tagged β subunit (52.7 kDa) of ATP synthase (TM_β-his_), which was subjected to Ni–NTA affinity purification within a linear range of binding capacity. Fractions of FF, WF, and EF were separated via SDS-PAGE and evaluated for presence of the his_6_-tagged β subunit via western blot with the his_6_ antibody (left). In order to determine the proportion of the his_6_-tagged β subunit in ATP synthase, TM_β-his_ was pretreated with 0.5% Triton X-100 prior to loading onto Ni–NTA resin. FF, WF, and EF were separated via SDS-PAGE and evaluated for his_6_-tag and β subunit by western blot with antibodies against the his_6_ and β subunit, respectively (middle and right). **B** TM prepared from Syn-AtpH contains the strep-tagged c subunit (8.8 kDa) of ATPase (TM_c-strep_), which was subjected to streptavidin-based affinity purification within linear range of binding capacity. Fractions of FF, WF, and EF were separated via SDS-PAGE and evaluated for the strep-tagged c-subunit via western blot with the anti-c antibody (left). In order to determine the proportion of strep-tagged c-subunit in ATP synthase, TM_c-strep_ was pretreated with 0.5% Triton X-100 prior to loading onto streptavidin resin. FF, WF, and EF were separated via SDS-PAGE and evaluated for the streptavidin tag and c-subunit through western blot with antibodies against streptavidin and the c-subunit, respectively (middle and right)

TM in the elution fraction (EF) was regarded as an F_1_-out vesicle with his_6_-tagged β, whereas TM in the flow-through fraction (FF) was regarded as an F_1_-in vesicle (Fig. 1A, left). Western blot analysis with the his_6_ antibody revealed approximately 74% of the signal from EF, suggesting that F_1_-out vesicles accounted for the majority of TM_β-his_. His_6_-tag-free vesicles with an F_1_-out orientation could be included in FF (Fig. 1A, left), although not detected. Accordingly, the amount of his_6_-tagged β-subunit incorporated into the ATP synthase of TM was determined. To this end, the TM was treated with Triton X-100 (0.5%) to expose the total β subunits prior to loading onto the Ni–NTA column. Most (96%) his_6_-tagged β-subunits were detected in the EF (Fig. 1A, middle). The minor (4%) signal from FF could be ascribed to the tagged subunit overflow because Ni–NTA resin was used at the maximum binding capacity. The same samples were analyzed via western blot using an anti-β antibody. Approximately the same signal intensities were detected in both FF and EF (Fig. 1A, right), suggesting that half of the β-subunits in F_1_-ATPase of TMs were his_6_-tagged.

To confirm these results, the N-terminus of the c-subunit of F_o_, which is exposed at the site opposite to the β subunit, was tagged with streptavidin. The recombinant strep-tagged c subunit was produced in Syn-AtpH (Table 1), and TM was prepared from the cells. The binding capacity of streptavidin resin for TM containing the strep-tagged c subunit exposed outside was determined as described above, which turned out to be around 1.2 µg Chl *a* per mL resin (Additional file 1: Fig. S1B).

The TM in FF was regarded as an F_o_-in vesicle, whereas the TM in EF was regarded as an F_o_-out vesicle with strep-tagged c (Fig. 1B, left). Western blotting with an anti-strep antibody revealed approximately 85% signal from FF, suggesting F_1_-out (F_o_-in) vesicles as the major portion of TM_c-strep_. This result also confirmed the above results obtained with the his_6_ tag, and more than three-quarters of TMs prepared from *Synechocystis* were in the orientation of F_1_-out (F_o_-in). Strep-tag-free vesicles were included in FF (Fig. 1B, left), although they were not detected. Accordingly, the extent of incorporation of the strep-tagged c-subunit into the ATP synthase of TM was determined. The TM was treated with Triton X-100 (0.5%) to expose the total c subunits prior to loading onto the streptavidin column. Most (94%) strep-tagged c-subunits were detected in the EF (Fig. 1B, middle). The minor (6%) signal from FF could also be ascribed to the tagged subunit overflow because streptavidin resin was used at the maximum binding capacity. The same samples were analyzed by western blotting using an anti-c antibody. Similarly, approximately the same signal intensities were detected in both FF and EF (Fig. 1B, right), suggesting that half of the c-subunits in the F_o_-ATPase of TM were strep-tagged.

### Enhancement of NADPH generation by removal of the F_1_ complex from ATP synthase

**Fig. 2:**
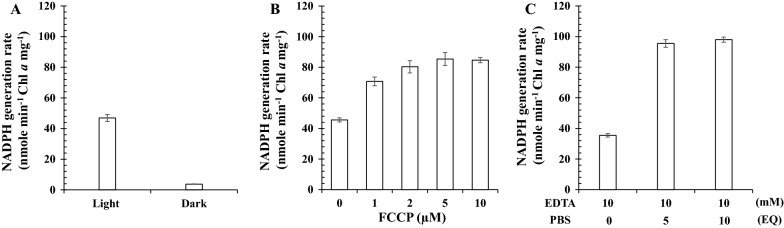
NADPH generation by pTM in the presence of FCCP and by the PBS-reconstituted TMe (pTMe). **A** pTM (5 μg Chl *a* mL^−1^) was mixed with TM-NADP^+^ buffer. The NADPH generation rate was determined under white light (50 μmol m^−2^ s^−1^) and in the dark. **B** The NADPH generation of pTM (5 μg Chl *a* mL^−1^) was measured in the presence of varying concentrations of FCCP (0–10 µM) under white light as described in (**A**). **C** TM (5 μg Chl *a* mL^−1^) was treated with 10 mM EDTA as described in the Materials and Methods. The resulting TMe was washed with TM buffer via ultracentrifugation at 150,000*g* and 4℃ for 1 h, followed by reconstitution for PBS with 5 and 10 EQs. The resulting pTMe was examined for NADPH generation as described in (**A**). One EQ of PBS consists of 3.6 µg mL^−1^ phycocyanin and 1.8 µg mL^−1^ allophycocyanin. All quantifications were independently repeated three times, and data are shown as the mean ± standard deviation (SD)

TM components can support the light-dependent generation of NADPH and ATP when NADP^+^ and ADP are present along with Fd and FNR [11, 13]. As anticipated, light-dependent generation of NADPH and ATP was observed with the purified TM (Additional file 1: Fig. S2). Fd and FNR were optimized to further enhance TM activity (Additional file 1: Fig. S3). The NADPH generation rate increased with the Fd concentration, but a higher amount of Fd (≥ 20 µM) inhibited this activity (Additional file 1: Fig. S3A). This result was in line with previous studies [13, 56], which showed that higher levels of Fd may lead to the accumulation of complexes between oxidized Fd and FNR-containing semiquinone FAD. An increase in FNR (1–2 µM) slightly enhanced activity (Additional file 1: Fig. S3B), implying that FNR is primarily associated with the purified TM. The PBS is a light-harvesting antenna that is easily lost during TM preparation (Additional file 1: Fig. S4 Lanes 1 and 2). To compensate for this loss, PBS was isolated and used for reconstitution of the purified TM. NADPH generation increased in a dose-dependent manner when reconstituted with up to 5 equivalents (EQs) of PBS (Additional file 1: Fig. S3C). One EQ of PBS was arbitrarily set as phycocyanin at 3.6 µg mL^−1^ and allophycocyanin at 1.8 µg mL^−1^ because purified PBS consists of phycocyanin and allophycocyanin at a ratio of approximately 2:1 (w/w). Western blot analysis demonstrated that PBS content was recovered up to 80–90% via reconstitution (Additional file 1: Fig. S4, lane 5). The rate of light-dependent NADPH generation by TM reconstituted with 5 EQs of PBS was approximately 45 nmol min^−1^ Chl *a* mg^−1^ (Fig. 2A) in the presence of 1 µM FNR and 10 µM Fd.

A previous study demonstrated that a high proton gradient of the TM inhibited the LEF [57]. Moreover, excess protons cause luminal acidification, which can downregulate photosynthetic electron transport [58]*.* Given the coupling of LEF with proton movement, disruption of this linkage would relieve the inhibitory effect of ΔpH on LEF. The protonophore FCCP was used to dissipate pmf [59]. As expected, the NADPH generation rate increased approximately two-fold in a dose-dependent manner when pTM (TM reconstituted with 5 EQs of PBS) was treated with up to 5 µM FCCP (Fig. 2B). A concomitant decline in ATP generation was observed (Additional file 1: Fig. S5). However, FCCP treatment may also trigger ROS generation [60, 61]. Therefore, other approaches to achieve this goal must be developed.

It was previously shown that cleavage of the F_1_ γ subunit of ATP synthase can lower the proton gradient by inducing proton leakage [62, 63]. Therefore, the F_1_ complex of ATP synthase was removed using EDTA. EDTA-sonic treatment was applied to the deprivation of F_1_ subunits [42]. An increase in the NADPH generation rate of approximately 35% was observed after treatment with 2.5 mM EDTA (Additional file 1: Fig. S6A). The residual subunits were examined via western blot analysis using antibodies against each subunit. The results revealed that F_1_ was deprived of approximately half of γ by increasing EDTA up to 10 mM, while more than 90% of the other subunits were removed (Additional file 1: Fig. S6B). As anticipated, ATP generation decreased after the same treatment (Additional file 1: Fig. S6C). No further enhancement of NADPH generation was observed at EDTA higher than 2.5 mM (Additional file 1: Fig. S6A).

Given the labile charge-charge interactions between PBS and TM [64], higher concentrations of EDTA could also remove PBS, as previously reported [65]. In fact, PBS of the EDTA (10 mM)-treated TM (TMe) was barely detected via western blot analysis using antibodies against the α subunits of allophycocyanin and phycocyanin (Additional file 1: Fig. S7 lane 1). Therefore, TMe was reconstituted with 5 and 10 EQs of PBS to yield pTMe, revealing an approximately five- to ten-fold increase in PBS content (Additional file 1: Fig. S7). The NADPH generation activity of pTMe reconstituted with 5 EQs was similar to that of pTMe with 10 EQs (Fig. 2C), which is approximately twice as large as that of TMe (10 mM EDTA without PBS reconstitution). Therefore, pTMe reconstituted with five EQs was used in subsequent experiments.

### Sustainability improvement via biosilicification

To protect the lipid bilayer and protein-pigment complexes of TM from external stress, they were kept in a defined space via coating with a rigid silica shield. Silaffin-derived peptide R5 (SSKKSGSYSGSKGSKRRIL) has been shown to mediate silicification, which can encapsulate proteins and bacteria to enhance stability and survivability [26, 46]. Inspired by this process, pTMe was biosilicified using R5 with a TMOS (Additional file 1: Fig. S8A and B). Biosilicified pTMe (b-pTMe) was fully precipitated via centrifugation even at 3000*g* for 1 min. Examination of NADPH generation activity of pTMe revealed that R5 and TMOS did not have any deleterious effects on NADPH generation (Additional file 1: Fig. S8A and B, white bars). pTMe could be biosilicified up to 40 µg Chl *a* mL^−1^ using R5 (0.5 mg mL^−1^) with TMOS (10 mM) (Additional file 1: Fig. S8C).

The fluorescence microscopy image of b-pTMe demonstrated an amorphous structure with a diameter in the range of 3 to 6 μm (Additional file 1: Fig. S9B), which was much larger than that of pTMe (Additional file 1: Fig. S9A). The Z-average of pTMe is approximately 160 nm (Additional file 1: Fig. S9C). As basic R5 is absorbed by pTMe, the surface charge increases in the positive direction. However, this was lowered back to the negative side when encapsulated with a silica shell by R5 (Additional file 1: Fig. S10), demonstrating surface changes caused by biosilicification.

**Fig. 3:**
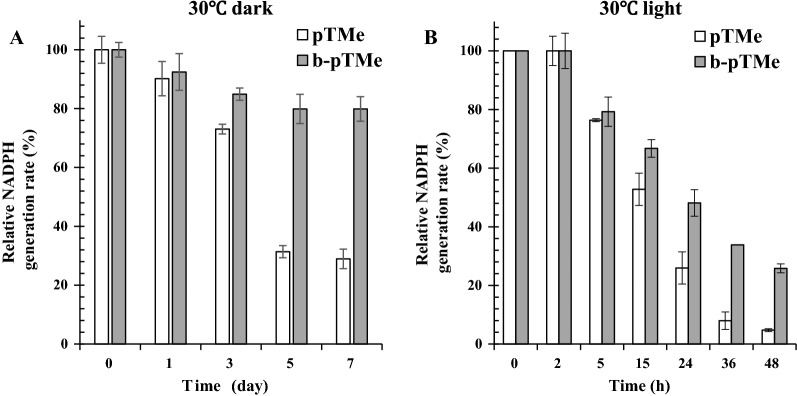
Sustainability of NADPH generation by pTMe and b-pTMe. pTMe and b-pTMe were kept in TM-NADP^+^ buffer at 30℃ for one week in dark (**A**) or for 48 h under white light (50 μmol m^−2^ s^−1^) **B**. Aliquots were withdrawn at time points indicated and briefly washed via ultracentrifugation (150,000*g* and 4℃ for 30 min). NADPH generation by pTMe and b-pTMe (each at 5 μg Chl *a* mL^−1^) were evaluated in TM-NADP^+^ buffer at 30℃ under white light (50 μmol m^−2^ s^−1^). The relative activities of pTMe and b-pTMe were represented as percentages of TM activity at time zero, which were virtually the same. All measurements were independently repeated three times, and data are shown as the mean ± standard deviation (SD)

We investigated whether the sustainability of pTMe was improved by biosilicification. pTMe and b-pTMe were placed in TM-NADP^+^ buffer at 30°C in the presence or absence of light, respectively. Approximately 80% of the NADPH generation activity of b-pTMe was preserved even after seven days in the dark, whereas only 30% activity was observed with pTMe under the same conditions (Fig. 3A). Thus, biosilicification improved the sustainability of NADPH generation. To investigate the matter further, levels of the four major components of LEF, PsbO (subunit of water splitting complex), PsbA (photosystem II protein D1), PC (luminal electron carrier), and PsaC (stromal subunit of photosystem I), were examined via western blot analysis. The four pTMe proteins exhibited decreases between days 3 and 7 (Additional file 1: Fig. S11A). Conversely, no less than 40% of each protein remained in b-pTMe for 7 days (Additional file 1: Fig. S11B). These results demonstrated the protective effect of the silica shell on NADPH generation.

NADPH generation activities of pTMe and b-pTMe in light decreased more drastically in two days compared with those in the dark (Fig. 3B), although biosilicification still exhibited a positive effect on NADPH generation. The four proteins involved in LEF were examined by western blotting, as described above. The results showed that fewer proteins remained after two days (Additional file 1: Fig. S12) compared to those after 3-day storage in the dark (Additional file 1: Fig. S11). This may be ascribed to the ROS generated under light, which can damage membrane lipids and protein [29], especially PsbA and PsbD in photosystem II [66].

General ROS formation by pTMe and b-pTMe in light was examined using the ROS indicator H_2_DCFDA. The cell lysate (32 µg) from *Synechocystis* was used as an esterase source to hydrolyze H_2_DCFDA (Additional file 1: Fig. S13A and B), and chloroform (4 µL) was used to lyse pTMe and b-pTMe in order to detect ROS formed inside the vesicle (Additional file 1: Fig. S13C and D). Singlet oxygen generated by pTMe and b-pTMe in light was also determined using SOSG. The singlet oxygen content was reduced by approximately 30% via biosilicification (Additional file 1: Fig. S14A), whereas general ROS formation was unaffected (Additional file 1: Fig. S14B). The -Si-O-Si- bond in silica [67] was found to be nonresponsive to ROS [68, 69]. Lysine (K) and arginine (R) were known to inhibit the oxidation of lipids and proteins by ROS [70, 71]. R5 (19 residues) contains four Ks and two Rs, which may possess antioxidative properties. Interestingly, R5 could quench singlet oxygen but did not scavenge superoxide or hydrogen peroxide (Additional file 1: Fig. S15). These results may account for the difference in singlet oxygen generation between pTMe and b-pTMe (Additional file 1: Fig. S14).

### Decrease in singlet oxygen generation through sustained LEF activity with Nox^m^

**Fig. 4:**
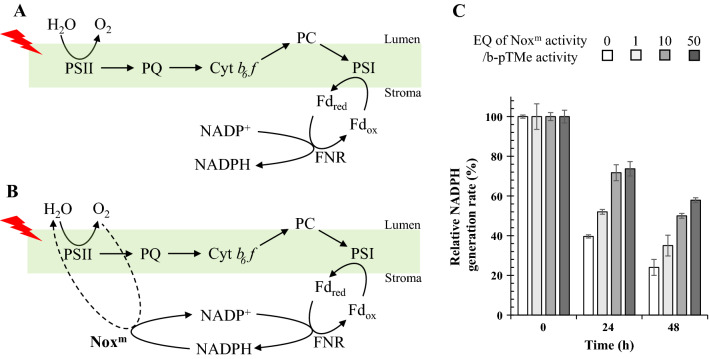
Effect of Nox^m^ on the sustainability of b-pTMe at 30℃ under light. **A** The LEF of TM is illustrated with its main components. **B** NADPH is oxidized to NADP^+^ by Nox^m^ with the simultaneous reduction of O_2_ to H_2_O. Thus, the cyclic conversion between NADP^+^ and NADPH was provided to keep LEF running. **C** Nox^m^ was added to b-pTMe at EQ ratios of 1:1, 10:1, and 50:1 in TM-NADP^+^ buffer, followed by incubation at 30℃ under white light (50 μmol m^−2^ s^−1^). b-pTMe without Nox^m^ was included as control. Aliquots were withdrawn at time points indicated and washed three times via centrifugation (at 3000*g* and 4℃ for 3 min). NADPH generation by b-pTMe (5 μg Chl *a* mL^−1^) was evaluated in TM-NADP^+^ buffer at 30℃ under white light (50 μmol m^−2^ s^−1^). The relative activities of b-pTMe were presented as percentages of those at time zero, which were virtually the same. All quantifications were independently repeated three times, and data are shown as the mean ± standard deviation (SD)

**Fig. 5:**
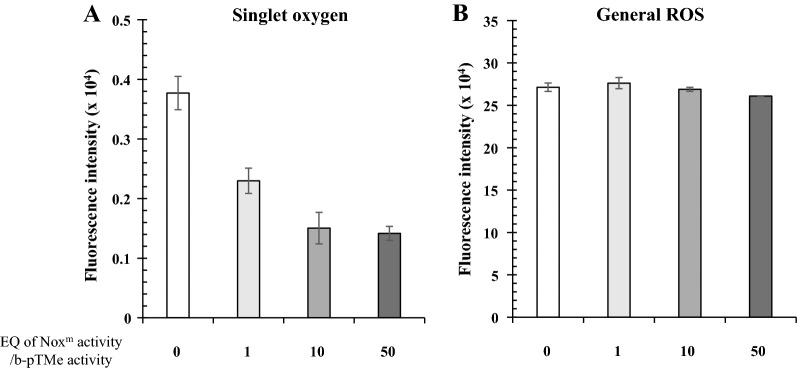
Effect of Nox^m^ on ROS generation of b-pTMe at 30℃ under light. **A** In order to detect singlet oxygen generated by b-pTMe, Singlet Oxygen Sensor Green (SOSG, 50 µM) was added to the reaction mixture, which contained b-pTMe at 5 µg Chl *a* mL^−1^ in TM-NADP^+^ buffer supplemented with varying EQ ratios of Nox^m^. Reaction without Nox^m^ was included as a control. The reaction was performed at 30℃ for 30 min under 50 μmol m^−2^ s^−1^ white light. Fluorescence from SOSG was detected at 525 nm with excitation at 504 nm after 30 min irradiation. **B** General ROS was detected with 0.2 mL aliquot withdrawn from reaction mixture of pTMe and b-pTMe at 5 µg Chl *a* mL^−1^ in TM-NADP^+^ buffer, which had been incubated at 30°C for 2 h under white light (50 μmol m^−2^ s^−1^). pTMe and b-pTMe were lysed with 4 μL chloroform, and 0.1 mM H_2_DCFDA was added to the reaction mixture with 32 µg *Synechocystis* cell lysate. The mixture was then incubated at 30°C for 10 min in dark, followed by detection of fluorescence at 525 nm after excitation at 488 nm. H_2_DCFDA without Nox^m^ was included as a control. All the quantifications were independently repeated three times, and data are shown as the mean ± standard deviation (SD)

If NADPH is not oxidized instantly after its generation, electron flow in the TM (Fig. 4A) ceases with electron carriers in more reduced states. Accumulation of NADPH can induce over-reduction of plastoquinone [30], which may have detrimental effects on the photosystem through the formation of ROS [30, 31]. To keep electron carriers in a less reduced state, cyclic conversion between NADPH and NADP^+^ was provided with an engineered water-forming NADPH oxidase (Nox^m^) (Fig. 4B), which was derived from the Nox of *Lactobacillus plantarum* after introducing mutations in G178R and L179R to obtain the substrate (i.e., coenzyme) preference for NADPH over NADH [34]. Nox^m^ was kinetically analyzed to illustrate activity of 18.1 ± 0.9 µmole min^−1^ mg^−1^ (Additional file 1: Fig. S16). The NADPH generation activity of pTMe (5 µg Chl *a* mL^−1^) was approximately 100 nmol min^−1^ Chl *a* mg^−1^ (Fig. 2C), which was not affected by biosilicification (Additional file 1: Fig. S8). Varying levels of Nox^m^ were mixed with the b-pTMe reaction mixture in the light for two days (Fig. 4C). b-pTMe without Nox^m^ was included as a control. The amount of Nox^m^ that could oxidize NADPH formed by b-pTMe was approximately 28 ng, which was arbitrarily set as 1 EQ. Aliquots were withdrawn at the indicated time points and washed via centrifugation to remove the Nox^m^. The absence of Nox^m^ after washing was confirmed through western blot with the anti-his_6_-tag antibody (data not shown). The introduction of Nox^m^ substantially improved the sustainability of NADPH generation in a ratio-dependent manner (Fig. 4C), which correlated with a decrease in singlet oxygen generation (Fig. 5A). Thus, retaining LEF activity under light results in the alleviation of the single oxygen generation of TM, which may be ascribed to the maintenance of electron carriers in a less reduced state with decreased exciton pressure at the reaction center of PS II [31]. However, Nox^m^ had no obvious effect on general ROS formation (Fig. 5B).

### Decrease in general ROS levels with catalase

**Fig. 6:**
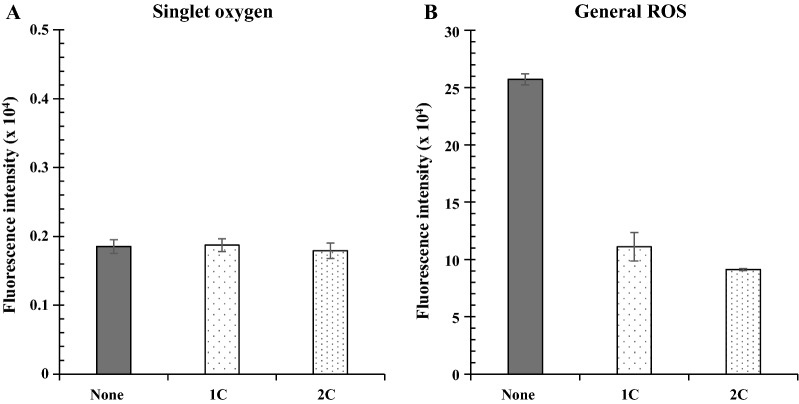
Effect of catalase on ROS generation by b-pTMe in the presence of Nox^m^. ROS generation was examined with b-pTMe (5 µg Chl *a* mL^−1^) in TM-NADP^+^ buffer supplemented with Nox^m^ (50 EQ ratios) and varying levels of catalase (1C, 37.5 µg mL^−1^; 2C, 75 µg mL^−1^). No addition of catalase (none) was included as a control condition. Singlet oxygen (**A**) and general ROS (**B**) were detected as described in Fig. 5. All quantifications were independently repeated three times, and data are shown as the mean ± standard deviation (SD)

**Fig. 7:**
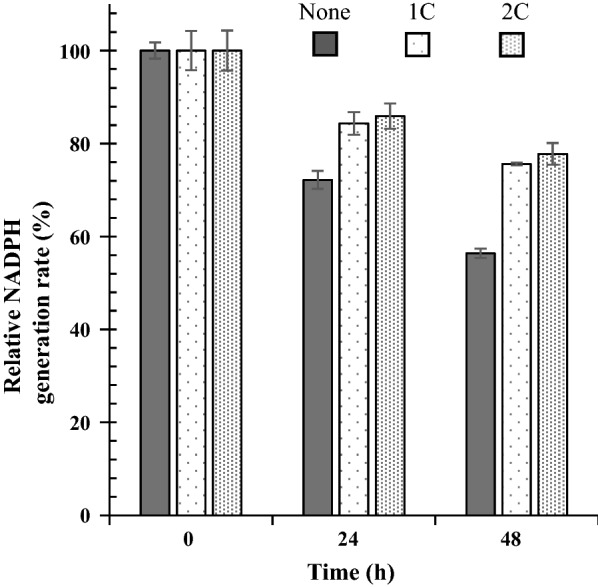
Sustainability of NADPH generation from b-pTMe in the presence of Nox^m^ and catalase. NADPH generation was examined with b-pTMe (5 µg Chl *a* mL^−1^) in TM-NAD^+^ buffer supplemented with Nox^m^ (50 EQ ratios) and varying levels of catalase (1C, 37.5 µg mL^−1^; 2C, 75 µg mL^−1^). The reaction mixture was incubated at 30℃ for 48 h under white light (50 μmol m^−2^ s^−1^). Aliquots were withdrawn at the indicated time points and washed three times via centrifugation (at 3000*g* and 4℃ for 3 min). NADPH generation by b-pTMe was evaluated in TM-NADP^+^ buffer at 30℃ under white light (50 μmol m^−2^ s^−1^). The relative activities of b-pTMe were represented as a percentage of those at time zero, which were virtually the same. All quantifications were independently repeated three times, and data are shown as the mean ± standard deviation (SD)

We examined whether the antioxidant enzymes SOD and catalase reduced the general ROS formed by b-pTMe. SOD did not exhibit any effect, but catalase lowered the general ROS to half the levels observed in the control (Additional file 1: Fig. S17). Once superoxide is formed by TM, it can be rapidly reduced to H_2_O_2_. It has been proposed that plastohydroquinone of the chloroplast TM can scavenge superoxide radicals to form H_2_O_2_ [72]. Catalase activity was used to examine the sustainability of b-pTMe in the presence of Nox^m^. General ROS levels were reduced to less than half through the addition of catalase (Fig. 6B), whereas singlet oxygen content was not affected (Fig. 6A). Consequently, approximately 80% of the NADPH production activity of b-pTMe was sustained in light for 48 h when catalase was added to the reaction mixture containing Nox^m^ (Fig. 7). Taken together, maintaining the NADPH utilization at no less than its formation and removing the hydrogen peroxide formed improved in vitro TM sustainability.

Practically, TM can be used for in vitro biosynthetic reactions requiring NADPH. If transhydrogenase [73] is provided, NADH can be readily produced from NADPH. Moreover, the total yield of a target material from the biosynthetic pathway may be elevated by increasing the amount of TM in the reaction mixture, for which the TM sustainability will be critical to keep the reaction rate constant. Furthermore, we intend to encapsulate Nox^m^, catalase, and pTMe together in a silica shell in a future study to examine the effects of clustering of the reaction mixture on the TM sustainability.

## Conclusion

Methods to improve the sustainability of in vitro NADPH generation by TMs have been extensively examined for improving vesicle utilization in biosynthetic reactions. The sustainability of NADPH generation in the TM can be extended via silica encapsulation. However, further removal of ROS by maintaining LEF in the presence of catalase is required to improve sustainability. Thus, if ROS formation by TM under light is properly suppressed or removed, it can be used to provide reducing power in vitro.

## Supplementary Information

**Additional file 1.** Sustainability of in vitro light-dependent NADPH generation by the thylakoid membrane of *Synechocystis* sp. PCC6803. Table S1. PCR primers used in this study. Fig. S1. Determination of binding capacity of Ni-NTA and streptavidin resins for TM_β-his_ and TM_c-strep_. Fig. S2. NADPH and ATP generation activities of TM in light and dark. Fig. S3. Optimization of FNR, Fd and phycobilisome (PBS) for the NADPH generation by TM. Fig. S4. Determination of PBS contents of cell-free extract, TM, and PBS-reconstituted TM (pTM). Fig. S5. ATP generation rate of pTM in the presence of FCCP. Fig. S6. Characterization of TM treated with varying concentrations of EDTA. Fig. S7. Determination of PBS contents of TM treated with 10 mM EDTA (TMe), and TMe reconstituted for PBS (pTMe). Fig. S8. Optimization of R5 and TMOS for the biosilicification of pTMe. Fig. S9. Microscopic observation of pTMe and b-pTMe. Fig. S10. Zeta potentials of pTMe, R5-pretreated pTMe, and b-pTMe. Fig. S11. Stability of the four major photosynthetic proteins of pTMe and b-pTMe during incubation at 30℃ in the dark. Fig. S12. Stability of the four major photosynthetic proteins of pTMe and b-pTMe during incubation at 30℃ in light. Fig. S13. Use of Synechocystis cell lysate for esterase activity and chloroform for lysis of pTMe and b-pTMe. Fig. S14. Determination of ROS generated by pTMe and b-pTMe. Fig. S15. Determination of antioxidant activity of R5. Fig. S16. Lineweaver-Burk plot (v_o_^-1^ vs. [NADPH]^-1^) illustrating the kinetic parameters of Nox^m^. Fig. S17. General ROS generated from b-pTMe at 30℃ in light in the presence of SOD and catalase.

## Data Availability

All data generated or analyzed during this study are included in this article and its additional files.
