# Supplementary material for: Sustainability of in vitro light-dependent NADPH generation by the thylakoid membrane of Synechocystis sp. PCC6803
Source: Microb Cell Fact. 2022 May 28;21:94. doi: 10.1186/s12934-022-01825-1 (PMC9148488; doi:10.1186/s12934-022-01825-1)
Supplement: Supplementary file 1 — Additional file 1. Sustainability of in vitro light-dependent NADPH generation by the thylakoid membrane of Synechocystis sp. PCC6803. Table S1. PCR primers used in this study. Fig. S1. Determination of binding capacity of Ni-NTA and streptavidin resins for TMβ-his and TMc-strep. Fig. S2. NADPH and ATP generation activities of TM in light and dark. Fig. S3. Optimization of FNR, Fd and phycobilisome (PBS) for the NADPH generation by TM. Fig. S4. Determination of PBS contents of cell-free extract, TM, and PBS-reconstituted TM (pTM). Fig. S5. ATP generation rate of pTM in the presence of FCCP. Fig. S6. Characterization of TM treated with varying concentrations of EDTA. Fig. S7. Determination of PBS contents of TM treated with 10 mM EDTA (TMe), and TMe reconstituted for PBS (pTMe). Fig. S8. Optimization of R5 and TMOS for the biosilicification of pTMe. Fig. S9. Microscopic observation of pTMe and b-pTMe. Fig. S10. Zeta potentials of pTMe, R5-pretreated pTMe, and b-pTMe. Fig. S11. Stability of the four major photosynthetic proteins of pTMe and b-pTMe during incubation at 30℃ in the dark. Fig. S12. Stability of the four major photosynthetic proteins of pTMe and b-pTMe during incubation at 30℃ in light. Fig. S13. Use of Synechocystis cell lysate for esterase activity and chloroform for lysis of pTMe and b-pTMe. Fig. S14. Determination of ROS generated by pTMe and b-pTMe. Fig. S15. Determination of antioxidant activity of R5. Fig. S16. Lineweaver-Burk plot (vo-1 vs. [NADPH]-1) illustrating the kinetic parameters of Noxm. Fig. S17. General ROS generated from b-pTMe at 30℃ in light in the presence of SOD and catalase. [file 12934_2022_1825_MOESM1_ESM.pptx]

## Slide 1
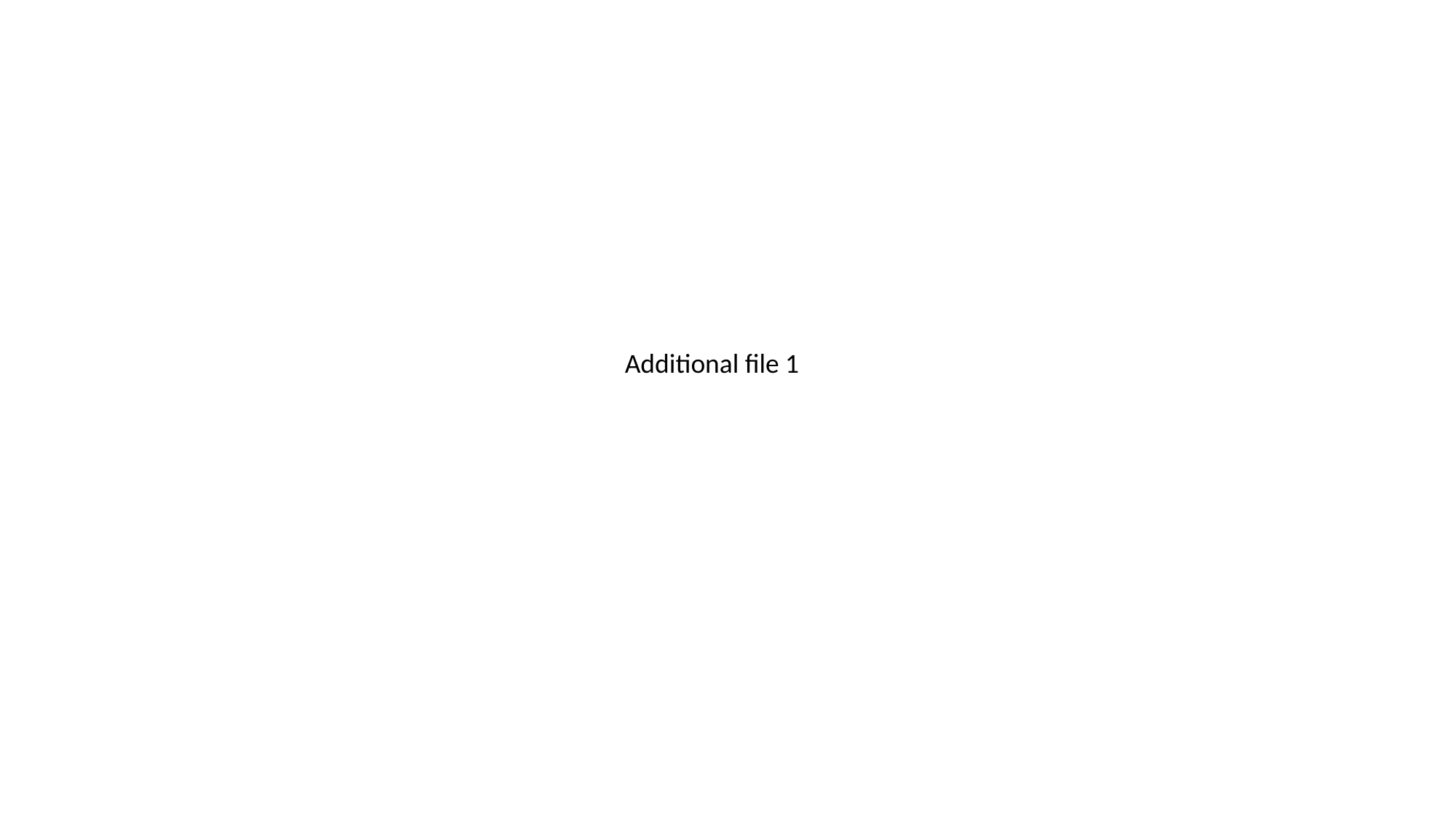

Additional file 1

## Slide 2
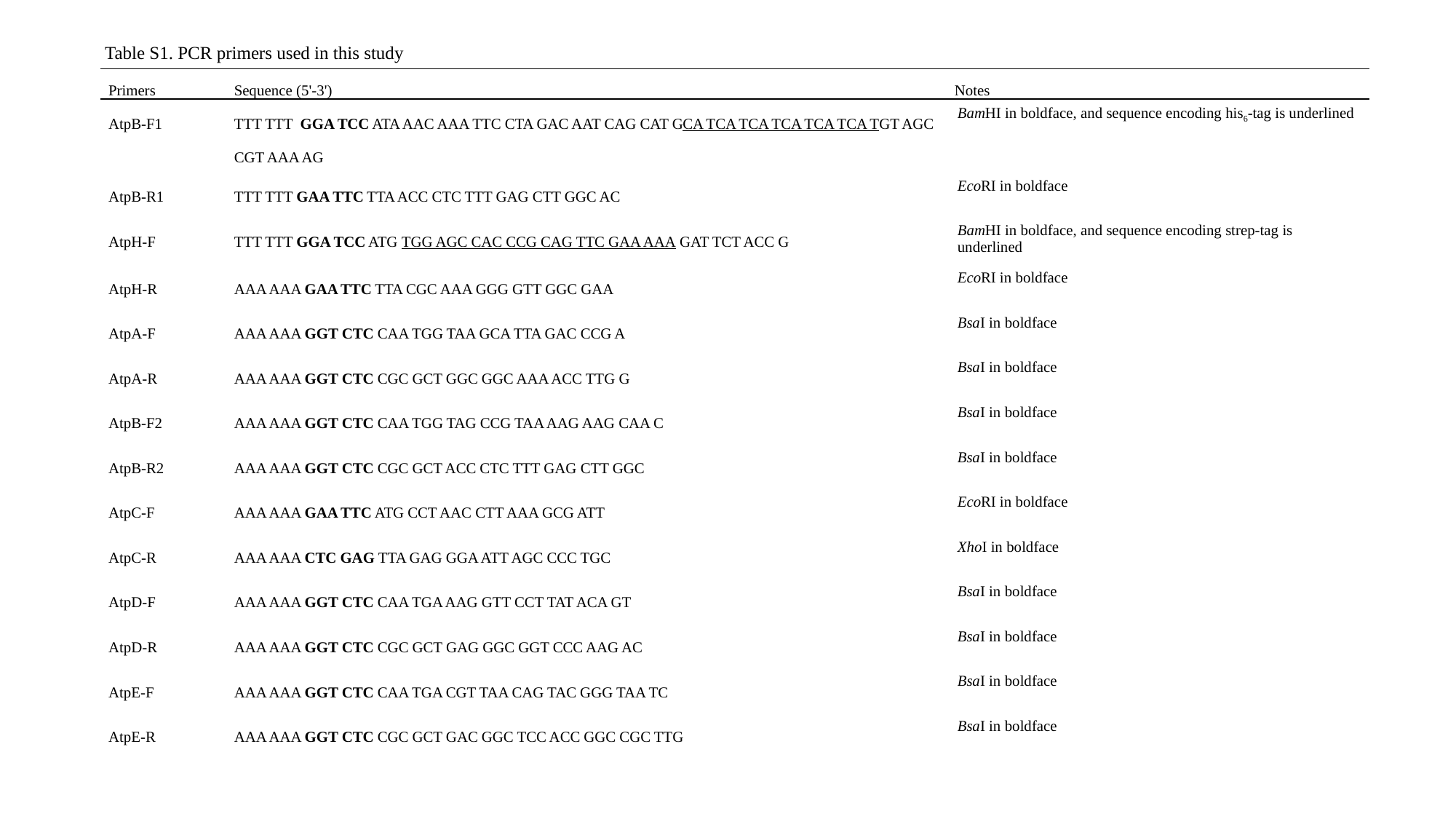

Table S1. PCR primers used in this study
| Primers | Sequence (5'-3') | Notes |
| --- | --- | --- |
| AtpB-F1 | TTT TTT GGA TCC ATA AAC AAA TTC CTA GAC AAT CAG CAT GCA TCA TCA TCA TCA TCA TGT AGC CGT AAA AG | BamHI in boldface, and sequence encoding his6-tag is underlined |
| AtpB-R1 | TTT TTT GAA TTC TTA ACC CTC TTT GAG CTT GGC AC | EcoRI in boldface |
| AtpH-F | TTT TTT GGA TCC ATG TGG AGC CAC CCG CAG TTC GAA AAA GAT TCT ACC G | BamHI in boldface, and sequence encoding strep-tag is underlined |
| AtpH-R | AAA AAA GAA TTC TTA CGC AAA GGG GTT GGC GAA | EcoRI in boldface |
| AtpA-F | AAA AAA GGT CTC CAA TGG TAA GCA TTA GAC CCG A | BsaI in boldface |
| AtpA-R | AAA AAA GGT CTC CGC GCT GGC GGC AAA ACC TTG G | BsaI in boldface |
| AtpB-F2 | AAA AAA GGT CTC CAA TGG TAG CCG TAA AAG AAG CAA C | BsaI in boldface |
| AtpB-R2 | AAA AAA GGT CTC CGC GCT ACC CTC TTT GAG CTT GGC | BsaI in boldface |
| AtpC-F | AAA AAA GAA TTC ATG CCT AAC CTT AAA GCG ATT | EcoRI in boldface |
| AtpC-R | AAA AAA CTC GAG TTA GAG GGA ATT AGC CCC TGC | XhoI in boldface |
| AtpD-F | AAA AAA GGT CTC CAA TGA AAG GTT CCT TAT ACA GT | BsaI in boldface |
| AtpD-R | AAA AAA GGT CTC CGC GCT GAG GGC GGT CCC AAG AC | BsaI in boldface |
| AtpE-F | AAA AAA GGT CTC CAA TGA CGT TAA CAG TAC GGG TAA TC | BsaI in boldface |
| AtpE-R | AAA AAA GGT CTC CGC GCT GAC GGC TCC ACC GGC CGC TTG | BsaI in boldface |

## Slide 3
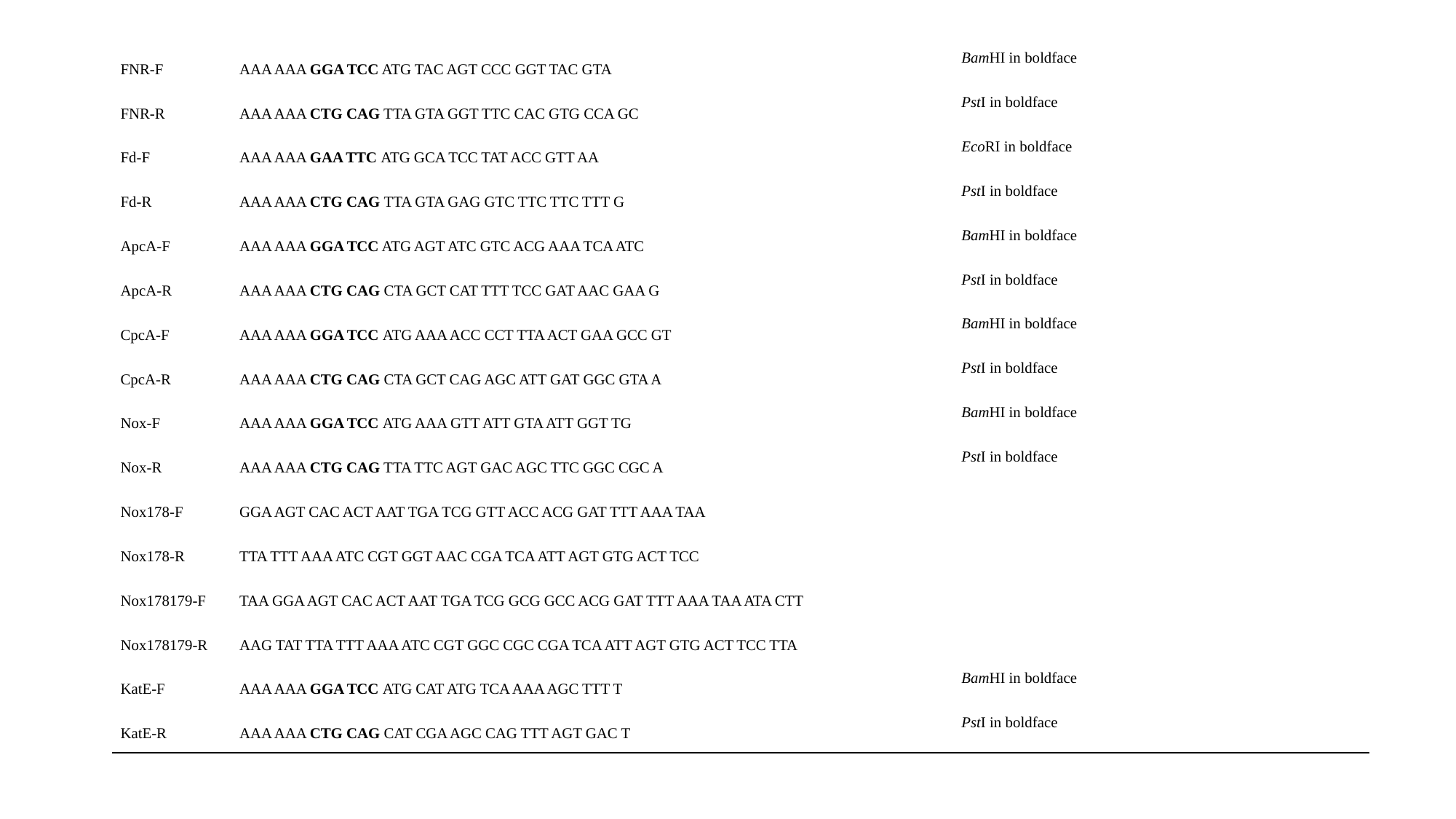

| FNR-F | AAA AAA GGA TCC ATG TAC AGT CCC GGT TAC GTA | BamHI in boldface |
| --- | --- | --- |
| FNR-R | AAA AAA CTG CAG TTA GTA GGT TTC CAC GTG CCA GC | PstI in boldface |
| Fd-F | AAA AAA GAA TTC ATG GCA TCC TAT ACC GTT AA | EcoRI in boldface |
| Fd-R | AAA AAA CTG CAG TTA GTA GAG GTC TTC TTC TTT G | PstI in boldface |
| ApcA-F | AAA AAA GGA TCC ATG AGT ATC GTC ACG AAA TCA ATC | BamHI in boldface |
| ApcA-R | AAA AAA CTG CAG CTA GCT CAT TTT TCC GAT AAC GAA G | PstI in boldface |
| CpcA-F | AAA AAA GGA TCC ATG AAA ACC CCT TTA ACT GAA GCC GT | BamHI in boldface |
| CpcA-R | AAA AAA CTG CAG CTA GCT CAG AGC ATT GAT GGC GTA A | PstI in boldface |
| Nox-F | AAA AAA GGA TCC ATG AAA GTT ATT GTA ATT GGT TG | BamHI in boldface |
| Nox-R | AAA AAA CTG CAG TTA TTC AGT GAC AGC TTC GGC CGC A | PstI in boldface |
| Nox178-F | GGA AGT CAC ACT AAT TGA TCG GTT ACC ACG GAT TTT AAA TAA | |
| Nox178-R | TTA TTT AAA ATC CGT GGT AAC CGA TCA ATT AGT GTG ACT TCC | |
| Nox178179-F | TAA GGA AGT CAC ACT AAT TGA TCG GCG GCC ACG GAT TTT AAA TAA ATA CTT | |
| Nox178179-R | AAG TAT TTA TTT AAA ATC CGT GGC CGC CGA TCA ATT AGT GTG ACT TCC TTA | |
| KatE-F | AAA AAA GGA TCC ATG CAT ATG TCA AAA AGC TTT T | BamHI in boldface |
| KatE-R | AAA AAA CTG CAG CAT CGA AGC CAG TTT AGT GAC T | PstI in boldface |

## Slide 4
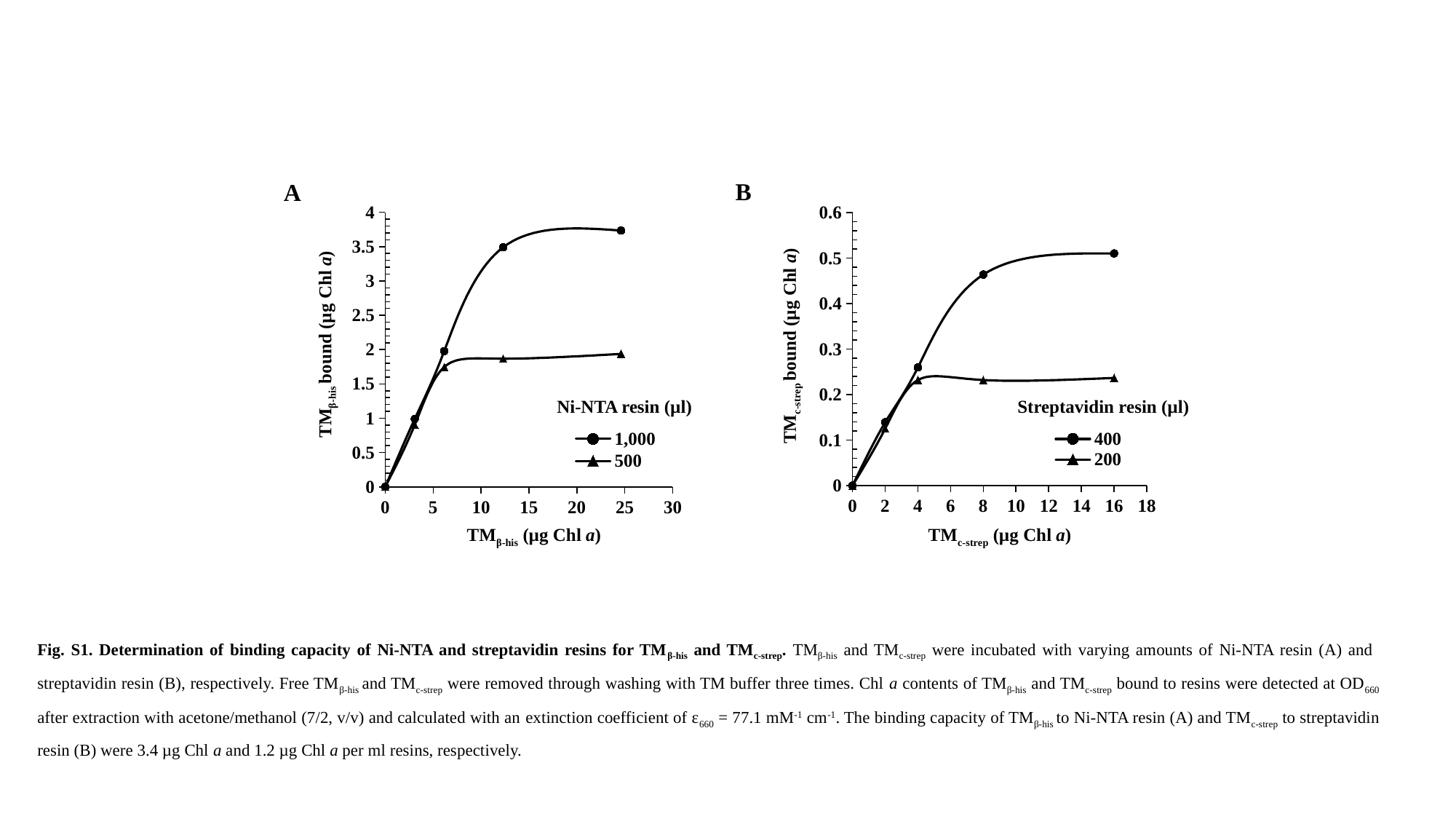

B
A
### Chart
| Category | 1,000 | 500 |
|---|---|---|
### Chart
| Category | 400 | 200 |
|---|---|---|TMβ-his bound (µg Chl a)
TMc-strep bound (µg Chl a)
Streptavidin resin (µl)
Ni-NTA resin (µl)
TMc-strep (µg Chl a)
TMβ-his (µg Chl a)
Fig. S1. Determination of binding capacity of Ni-NTA and streptavidin resins for TMβ-his and TMc-strep. TMβ-his and TMc-strep were incubated with varying amounts of Ni-NTA resin (A) and streptavidin resin (B), respectively. Free TMβ-his and TMc-strep were removed through washing with TM buffer three times. Chl a contents of TMβ-his and TMc-strep bound to resins were detected at OD660 after extraction with acetone/methanol (7/2, v/v) and calculated with an extinction coefficient of ε660 = 77.1 mM-1 cm-1. The binding capacity of TMβ-his to Ni-NTA resin (A) and TMc-strep to streptavidin resin (B) were 3.4 µg Chl a and 1.2 µg Chl a per ml resins, respectively.

## Slide 5
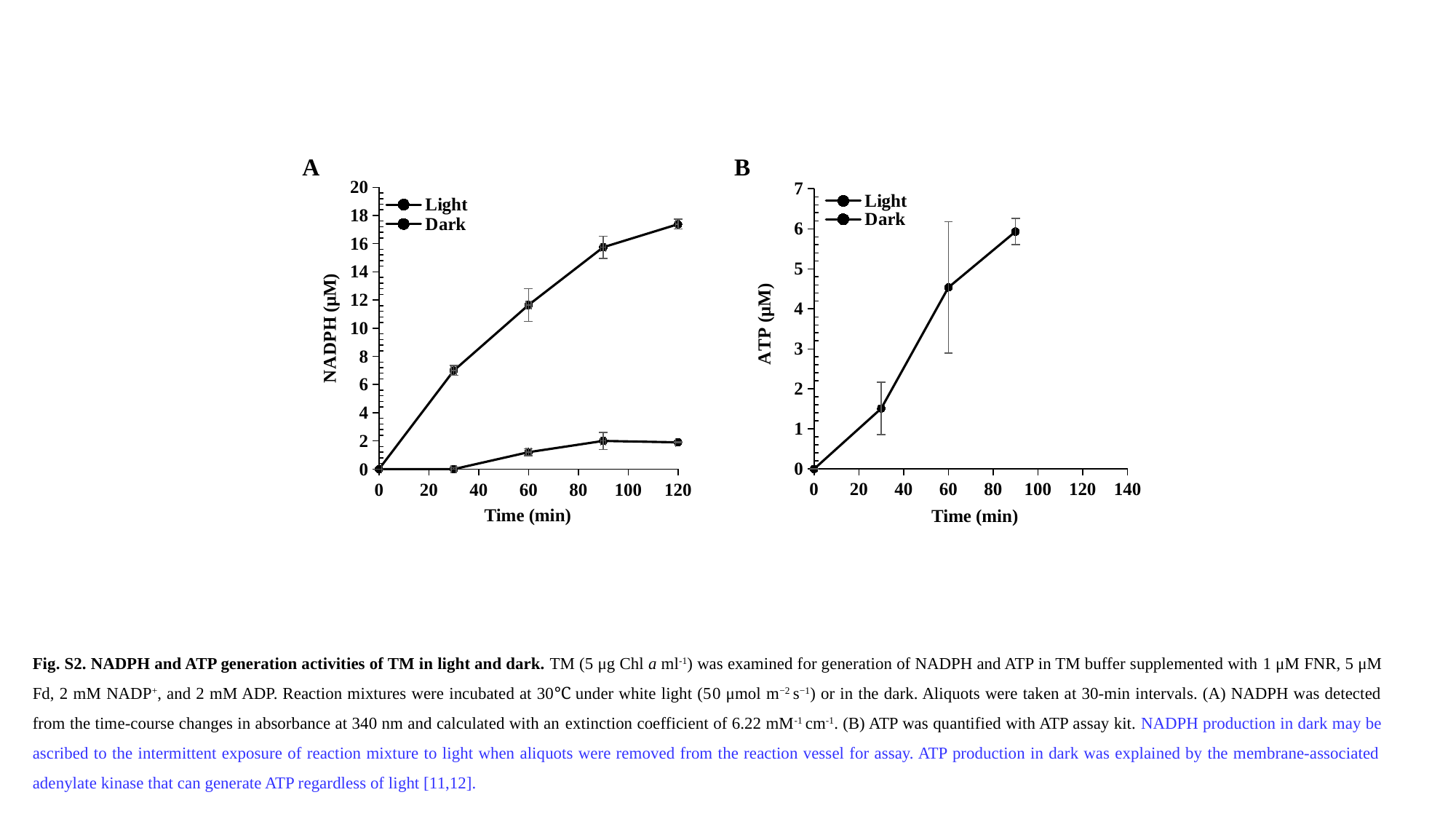

A
B
### Chart
| Category | Light | Dark |
|---|---|---|
### Chart
| Category | Light | Dark |
|---|---|---|Time (min)
Time (min)
Fig. S2. NADPH and ATP generation activities of TM in light and dark. TM (5 μg Chl a ml-1) was examined for generation of NADPH and ATP in TM buffer supplemented with 1 μM FNR, 5 μM Fd, 2 mM NADP+, and 2 mM ADP. Reaction mixtures were incubated at 30℃ under white light (50 μmol m−2 s−1) or in the dark. Aliquots were taken at 30-min intervals. (A) NADPH was detected from the time-course changes in absorbance at 340 nm and calculated with an extinction coefficient of 6.22 mM-1 cm-1. (B) ATP was quantified with ATP assay kit. NADPH production in dark may be ascribed to the intermittent exposure of reaction mixture to light when aliquots were removed from the reaction vessel for assay. ATP production in dark was explained by the membrane-associated adenylate kinase that can generate ATP regardless of light [11,12].

## Slide 6
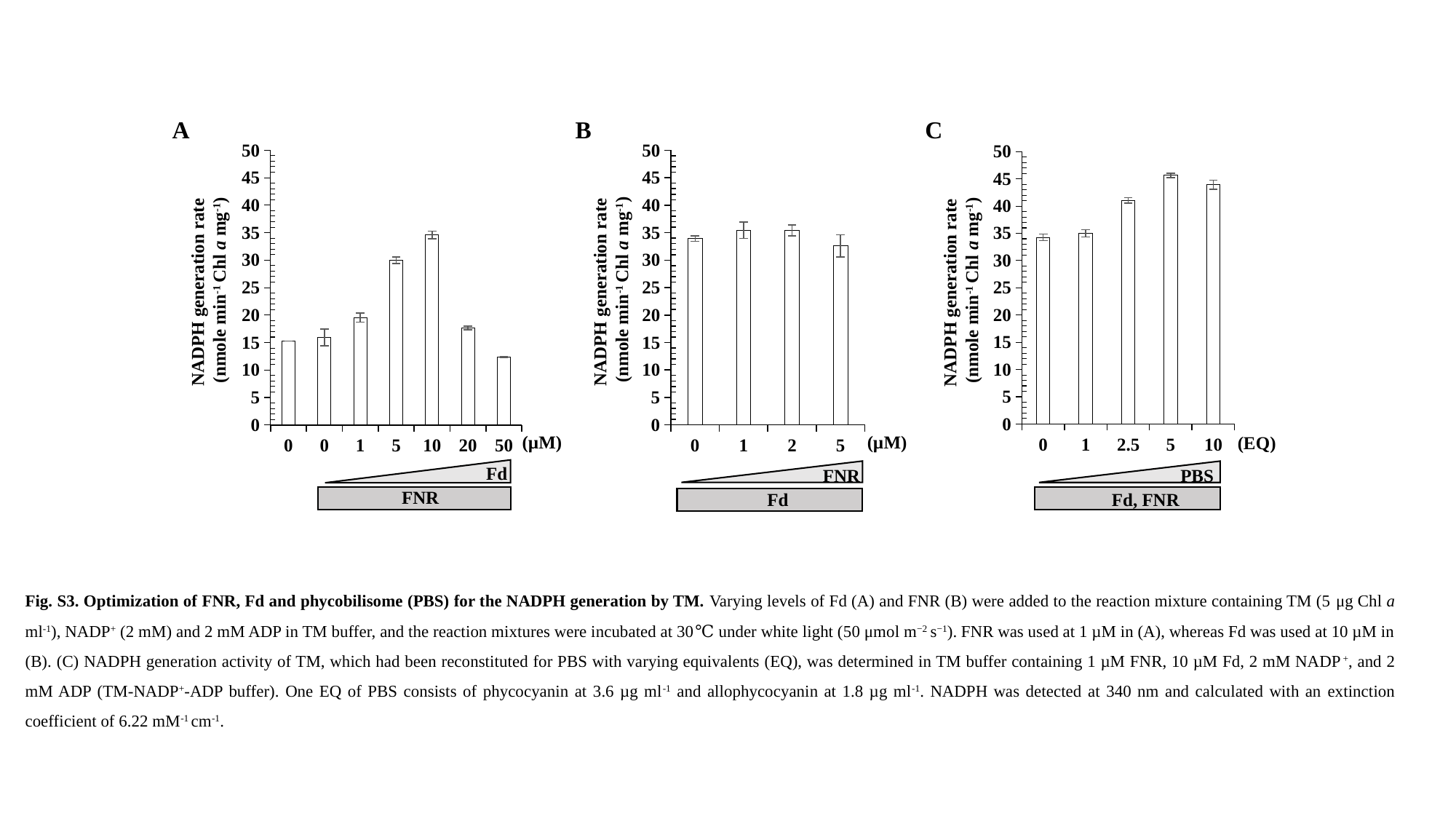

C
A
B
### Chart
| Category | |
|---|---|
| 0 | 15.29743912678424 |
| 0 | 15.93660401876837 |
| 1 | 19.55855947757936 |
| 5 | 29.99831344709342 |
| 10 | 34.58066071534478 |
| 20 | 17.64105364644413 |
| 50 | 12.31464855995737 |
### Chart
| Category | |
|---|---|
| 0 | 34.25 |
| 1 | 35.0 |
| 2.5 | 41.0344827586207 |
| 5 | 45.63218390804595 |
| 10 | 43.90804597701149 |
### Chart
| Category | |
|---|---|
| 0 | 33.91684314112 |
| 1 | 35.4355077593791 |
| 2 | 35.43550775937909 |
| 5 | 32.59308735087276 |NADPH generation rate
 (nmole min-1 Chl a mg-1)
NADPH generation rate
 (nmole min-1 Chl a mg-1)
NADPH generation rate
 (nmole min-1 Chl a mg-1)
(µM)
(µM)
(EQ)
Fd
FNR
PBS
FNR
Fd
Fd, FNR
Fig. S3. Optimization of FNR, Fd and phycobilisome (PBS) for the NADPH generation by TM. Varying levels of Fd (A) and FNR (B) were added to the reaction mixture containing TM (5 μg Chl a ml-1), NADP+ (2 mM) and 2 mM ADP in TM buffer, and the reaction mixtures were incubated at 30℃ under white light (50 μmol m−2 s−1). FNR was used at 1 µM in (A), whereas Fd was used at 10 µM in (B). (C) NADPH generation activity of TM, which had been reconstituted for PBS with varying equivalents (EQ), was determined in TM buffer containing 1 µM FNR, 10 µM Fd, 2 mM NADP+, and 2 mM ADP (TM-NADP+-ADP buffer). One EQ of PBS consists of phycocyanin at 3.6 µg ml-1 and allophycocyanin at 1.8 µg ml-1. NADPH was detected at 340 nm and calculated with an extinction coefficient of 6.22 mM-1 cm-1.

## Slide 7
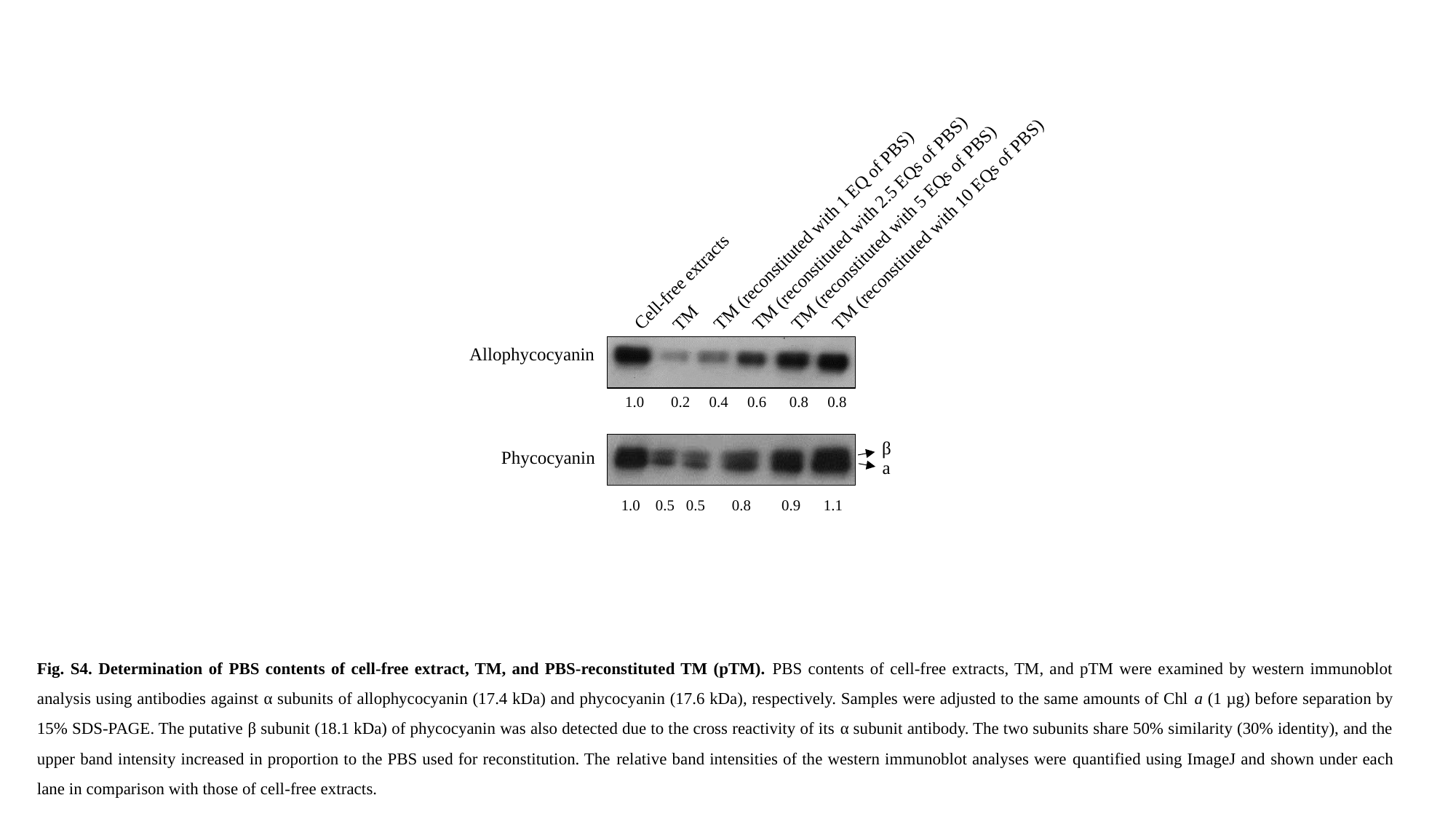

TM (reconstituted with 5 EQs of PBS)
TM (reconstituted with 2.5 EQs of PBS)
TM (reconstituted with 10 EQs of PBS)
TM (reconstituted with 1 EQ of PBS)
TM
Cell-free extracts
Allophycocyanin
 1.0 0.2 0.4 0.6 0.8 0.8
β
Phycocyanin
a
 1.0 0.5 0.5 0.8 0.9 1.1
Fig. S4. Determination of PBS contents of cell-free extract, TM, and PBS-reconstituted TM (pTM). PBS contents of cell-free extracts, TM, and pTM were examined by western immunoblot analysis using antibodies against α subunits of allophycocyanin (17.4 kDa) and phycocyanin (17.6 kDa), respectively. Samples were adjusted to the same amounts of Chl a (1 µg) before separation by 15% SDS-PAGE. The putative β subunit (18.1 kDa) of phycocyanin was also detected due to the cross reactivity of its α subunit antibody. The two subunits share 50% similarity (30% identity), and the upper band intensity increased in proportion to the PBS used for reconstitution. The relative band intensities of the western immunoblot analyses were quantified using ImageJ and shown under each lane in comparison with those of cell-free extracts.

## Slide 8
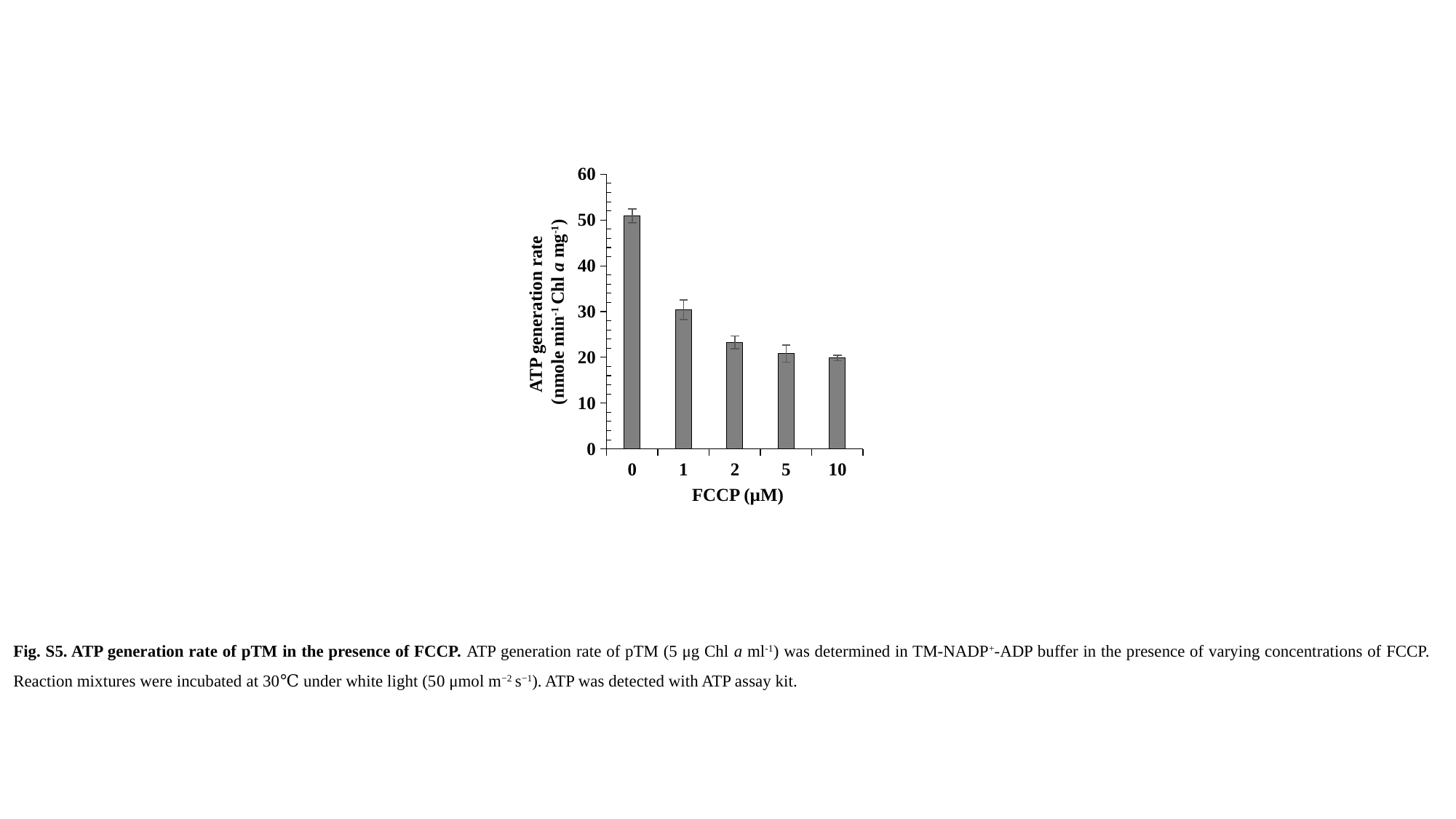

### Chart
| Category | |
|---|---|
| 0 | 50.9 |
| 1 | 30.414 |
| 2 | 23.290000000000003 |
| 5 | 20.824 |
| 10 | 19.865000000000002 |ATP generation rate
 (nmole min-1 Chl a mg-1)
FCCP (µM)
Fig. S5. ATP generation rate of pTM in the presence of FCCP. ATP generation rate of pTM (5 μg Chl a ml-1) was determined in TM-NADP+-ADP buffer in the presence of varying concentrations of FCCP. Reaction mixtures were incubated at 30℃ under white light (50 μmol m−2 s−1). ATP was detected with ATP assay kit.

## Slide 9
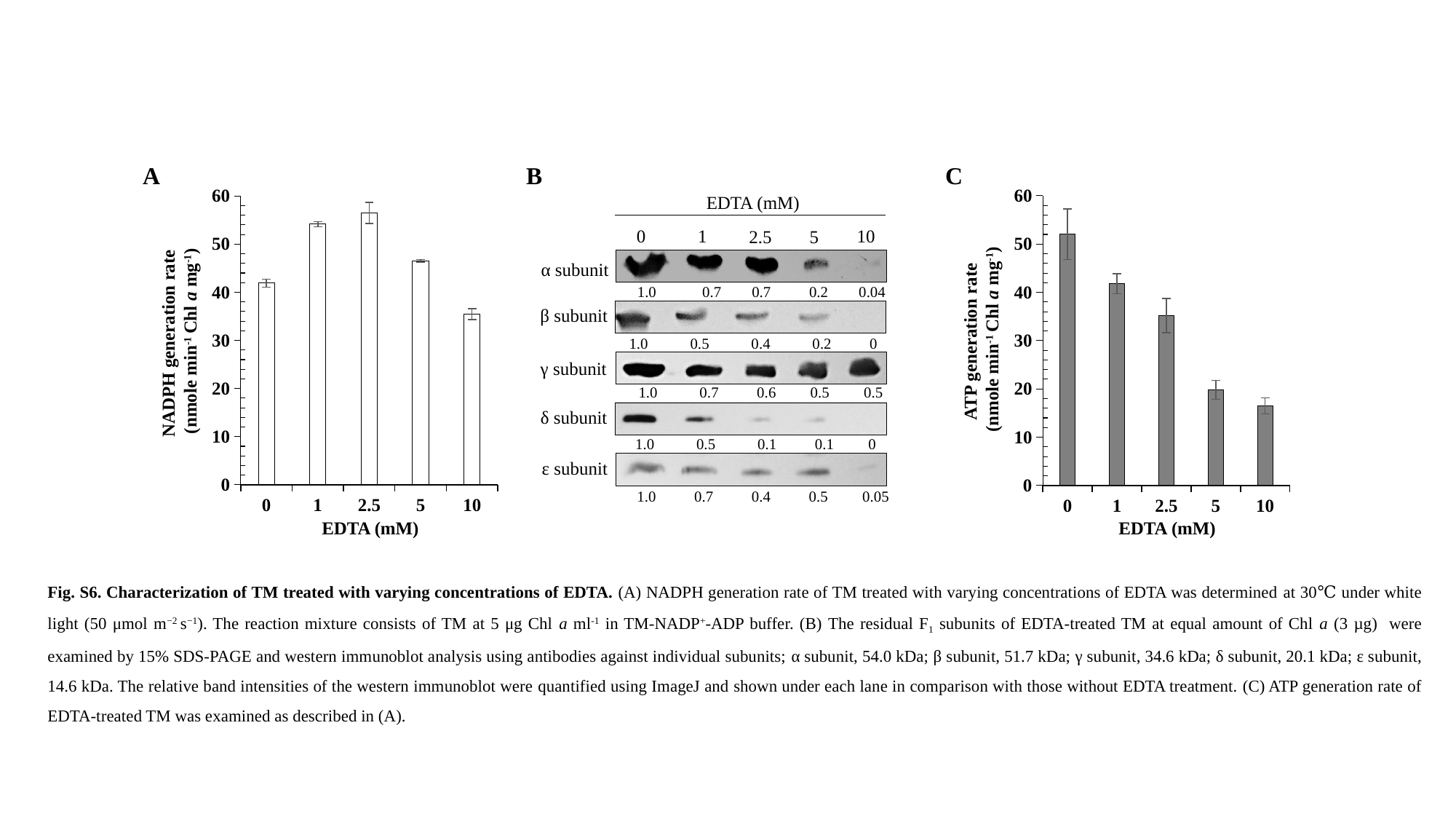

B
C
A
### Chart
| Category | |
|---|---|
| 0 | 41.8833333333333 |
| 1 | 54.13333333333334 |
| 2.5 | 56.46666666666663 |
| 5 | 46.54999999999998 |
| 10 | 35.46666666666666 |
### Chart
| Category | |
|---|---|
| 0 | 52.03 |
| 1 | 41.8 |
| 2.5 | 35.2 |
| 5 | 19.8 |
| 10 | 16.5 | EDTA (mM)
0
1
10
2.5
5
α subunit
β subunit
γ subunit
δ subunit
ε subunit
1.0 0.7 0.7 0.2 0.04
ATP generation rate
 (nmole min-1 Chl a mg-1)
NADPH generation rate
 (nmole min-1 Chl a mg-1)
1.0 0.5 0.4 0.2 0
1.0 0.7 0.6 0.5 0.5
1.0 0.5 0.1 0.1 0
1.0 0.7 0.4 0.5 0.05
EDTA (mM)
EDTA (mM)
Fig. S6. Characterization of TM treated with varying concentrations of EDTA. (A) NADPH generation rate of TM treated with varying concentrations of EDTA was determined at 30℃ under white light (50 μmol m−2 s−1). The reaction mixture consists of TM at 5 μg Chl a ml-1 in TM-NADP+-ADP buffer. (B) The residual F1 subunits of EDTA-treated TM at equal amount of Chl a (3 µg) were examined by 15% SDS-PAGE and western immunoblot analysis using antibodies against individual subunits; α subunit, 54.0 kDa; β subunit, 51.7 kDa; γ subunit, 34.6 kDa; δ subunit, 20.1 kDa; ε subunit, 14.6 kDa. The relative band intensities of the western immunoblot were quantified using ImageJ and shown under each lane in comparison with those without EDTA treatment. (C) ATP generation rate of EDTA-treated TM was examined as described in (A).

## Slide 10
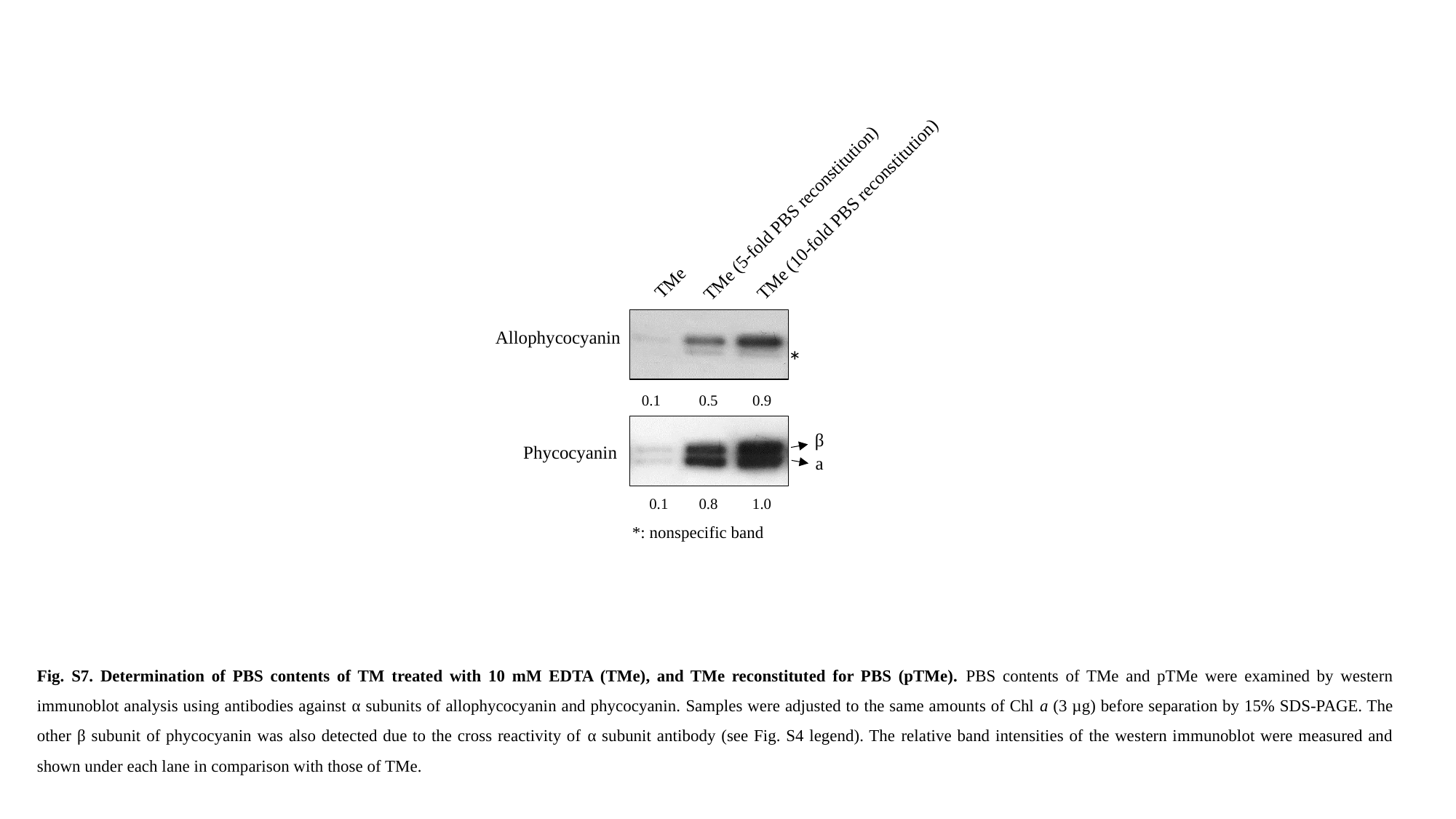

TMe (5-fold PBS reconstitution)
TMe (10-fold PBS reconstitution)
TMe
Allophycocyanin
*
 0.1 0.5 0.9
β
Phycocyanin
a
 0.1 0.8 1.0
*: nonspecific band
Fig. S7. Determination of PBS contents of TM treated with 10 mM EDTA (TMe), and TMe reconstituted for PBS (pTMe). PBS contents of TMe and pTMe were examined by western immunoblot analysis using antibodies against α subunits of allophycocyanin and phycocyanin. Samples were adjusted to the same amounts of Chl a (3 µg) before separation by 15% SDS-PAGE. The other β subunit of phycocyanin was also detected due to the cross reactivity of α subunit antibody (see Fig. S4 legend). The relative band intensities of the western immunoblot were measured and shown under each lane in comparison with those of TMe.

## Slide 11
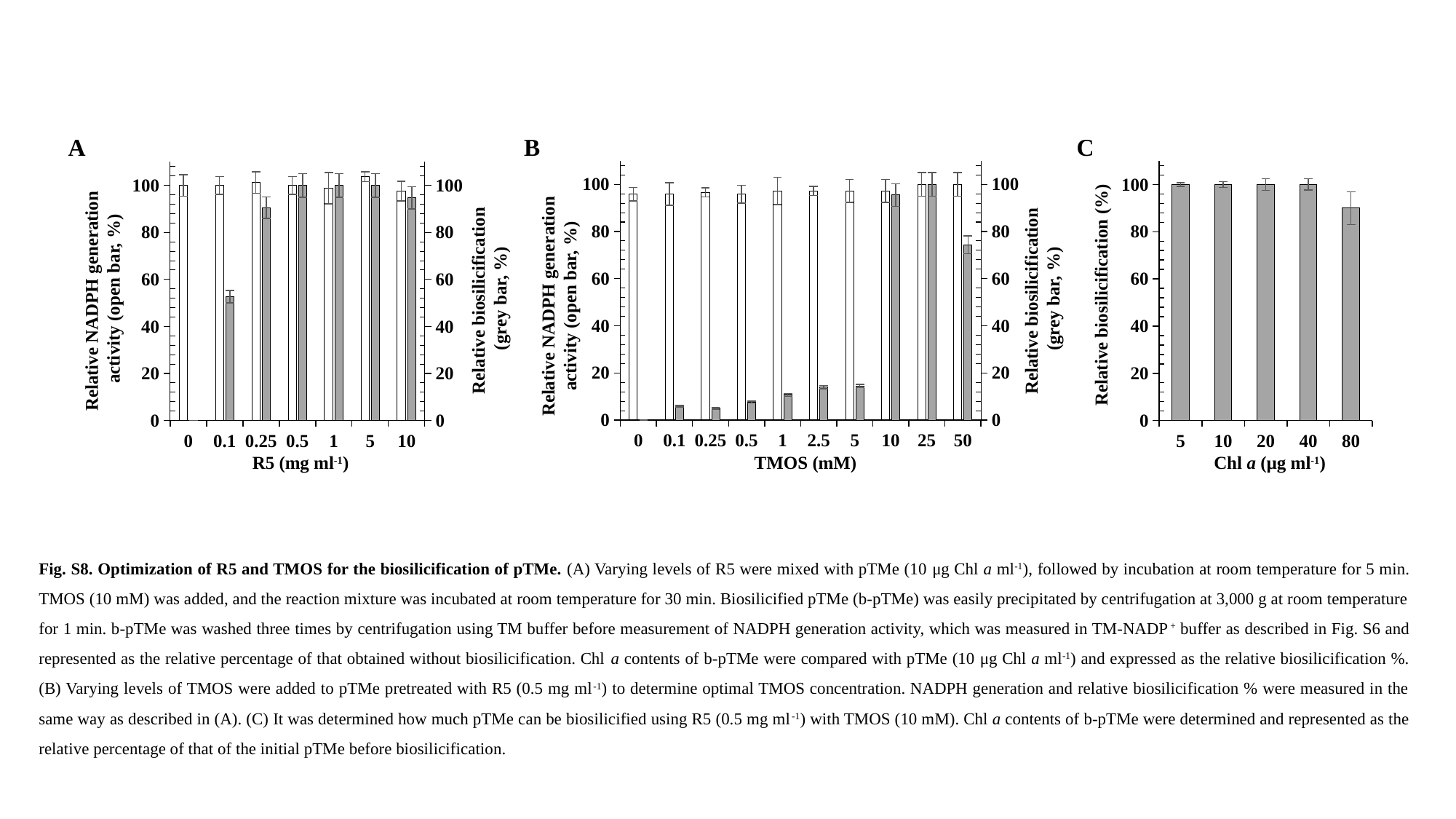

A
B
C
### Chart
| Category | |
|---|---|
| 5 | 100.0 |
| 10 | 100.0 |
| 20 | 100.0 |
| 40 | 100.0 |
| 80 | 90.0 |
### Chart
| Category | tCMV activity | biosilicificated tCMV | tCMV activity | biosilicificated tCMV |
|---|---|---|---|---|
| 0 | 95.83333333333333 | 0.0 | 95.83333333333333 | 0.0 |
| 0.1 | 95.83333333333333 | 5.85299455535388 | 95.83333333333333 | 5.85299455535388 |
| 0.25 | 96.52777777777777 | 4.945553539019967 | 96.52777777777777 | 4.945553539019967 |
| 0.5 | 95.83333333333333 | 7.667876588021757 | 95.83333333333333 | 7.667876588021757 |
| 1 | 97.22222222222224 | 10.61705989110706 | 97.22222222222224 | 10.61705989110706 |
| 2.5 | 97.22222222222224 | 14.01996370235935 | 97.22222222222224 | 14.01996370235935 |
| 5 | 97.22222222222224 | 14.4736842105263 | 97.22222222222224 | 14.4736842105263 |
| 10 | 97.22222222222224 | 95.4627949183303 | 97.22222222222224 | 95.4627949183303 |
| 25 | 100.0 | 100.0 | 100.0 | 100.0 |
| 50 | 100.0 | 74.36479128856624 | 100.0 | 74.36479128856624 |
### Chart
| Category | tCMV activity | biosilicificated tCMV | tCMV activity | biosilicificated tCMV |
|---|---|---|---|---|
| 0 | 100.0 | 0.0 | 100.0 | 0.0 |
| 0.1 | 100.0 | 52.63157894736842 | 100.0 | 52.63157894736842 |
| 0.25 | 101.2077294685992 | 90.52631578947367 | 101.2077294685992 | 90.52631578947367 |
| 0.5 | 100.0 | 100.0 | 100.0 | 100.0 |
| 1 | 98.79227053140069 | 100.0 | 98.79227053140069 | 100.0 |
| 5 | 103.623188405797 | 100.0 | 103.623188405797 | 100.0 |
| 10 | 97.58454106280206 | 94.73684210526315 | 97.58454106280206 | 94.73684210526315 |Relative biosilicification
 (grey bar, %)
Relative NADPH generation
 activity (open bar, %)
Relative biosilicification
 (grey bar, %)
Relative biosilicification (%)
Relative NADPH generation activity (open bar, %)
TMOS (mM)
R5 (mg ml-1)
Chl a (µg ml-1)
Fig. S8. Optimization of R5 and TMOS for the biosilicification of pTMe. (A) Varying levels of R5 were mixed with pTMe (10 μg Chl a ml-1), followed by incubation at room temperature for 5 min. TMOS (10 mM) was added, and the reaction mixture was incubated at room temperature for 30 min. Biosilicified pTMe (b-pTMe) was easily precipitated by centrifugation at 3,000 g at room temperature for 1 min. b-pTMe was washed three times by centrifugation using TM buffer before measurement of NADPH generation activity, which was measured in TM-NADP+ buffer as described in Fig. S6 and represented as the relative percentage of that obtained without biosilicification. Chl a contents of b-pTMe were compared with pTMe (10 μg Chl a ml-1) and expressed as the relative biosilicification %. (B) Varying levels of TMOS were added to pTMe pretreated with R5 (0.5 mg ml-1) to determine optimal TMOS concentration. NADPH generation and relative biosilicification % were measured in the same way as described in (A). (C) It was determined how much pTMe can be biosilicified using R5 (0.5 mg ml-1) with TMOS (10 mM). Chl a contents of b-pTMe were determined and represented as the relative percentage of that of the initial pTMe before biosilicification.

## Slide 12
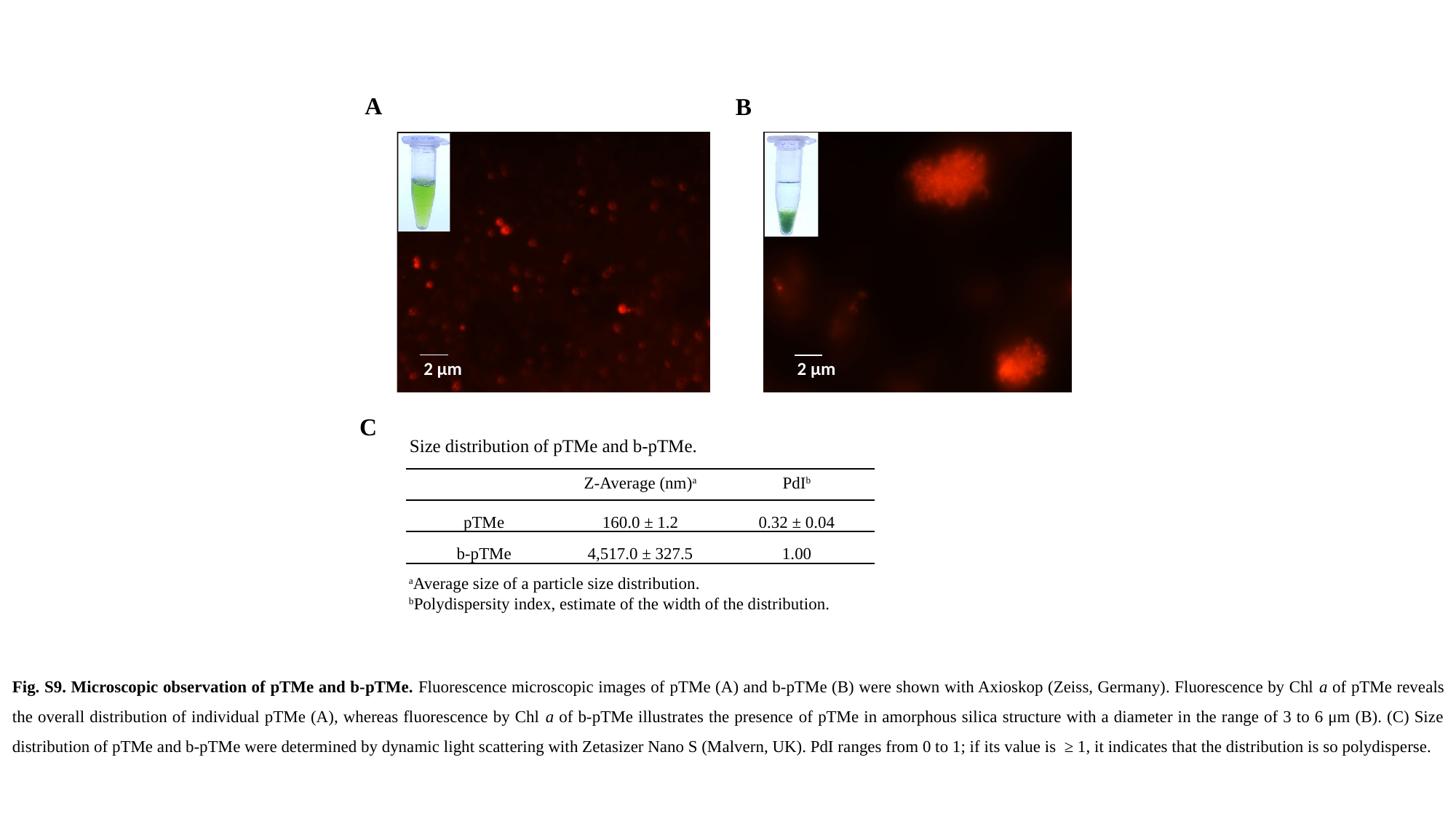

A
B
2 µm
2 µm
C
Size distribution of pTMe and b-pTMe.
| | Z-Average (nm)a | PdIb |
| --- | --- | --- |
| pTMe | 160.0 ± 1.2 | 0.32 ± 0.04 |
| b-pTMe | 4,517.0 ± 327.5 | 1.00 |
aAverage size of a particle size distribution.
bPolydispersity index, estimate of the width of the distribution.
Fig. S9. Microscopic observation of pTMe and b-pTMe. Fluorescence microscopic images of pTMe (A) and b-pTMe (B) were shown with Axioskop (Zeiss, Germany). Fluorescence by Chl a of pTMe reveals the overall distribution of individual pTMe (A), whereas fluorescence by Chl a of b-pTMe illustrates the presence of pTMe in amorphous silica structure with a diameter in the range of 3 to 6 μm (B). (C) Size distribution of pTMe and b-pTMe were determined by dynamic light scattering with Zetasizer Nano S (Malvern, UK). PdI ranges from 0 to 1; if its value is ≥ 1, it indicates that the distribution is so polydisperse.

## Slide 13
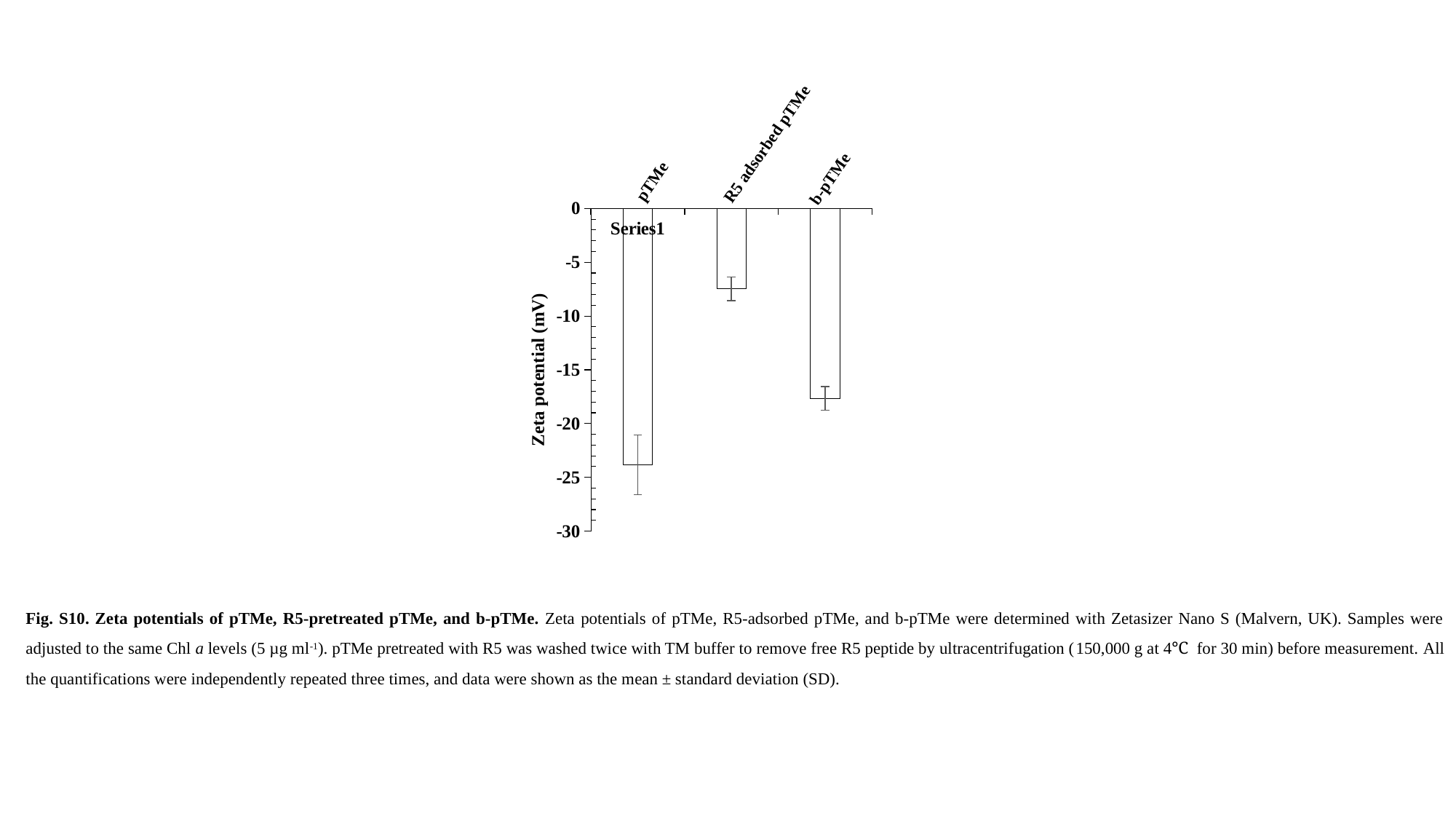

R5 adsorbed pTMe
b-pTMe
pTMe
### Chart
| Category | |
|---|---|
| | -23.83333333333332 |
| | -7.475 |
| | -17.66666666666667 |Zeta potential (mV)
Fig. S10. Zeta potentials of pTMe, R5-pretreated pTMe, and b-pTMe. Zeta potentials of pTMe, R5-adsorbed pTMe, and b-pTMe were determined with Zetasizer Nano S (Malvern, UK). Samples were adjusted to the same Chl a levels (5 µg ml-1). pTMe pretreated with R5 was washed twice with TM buffer to remove free R5 peptide by ultracentrifugation (150,000 g at 4℃ for 30 min) before measurement. All the quantifications were independently repeated three times, and data were shown as the mean ± standard deviation (SD).

## Slide 14
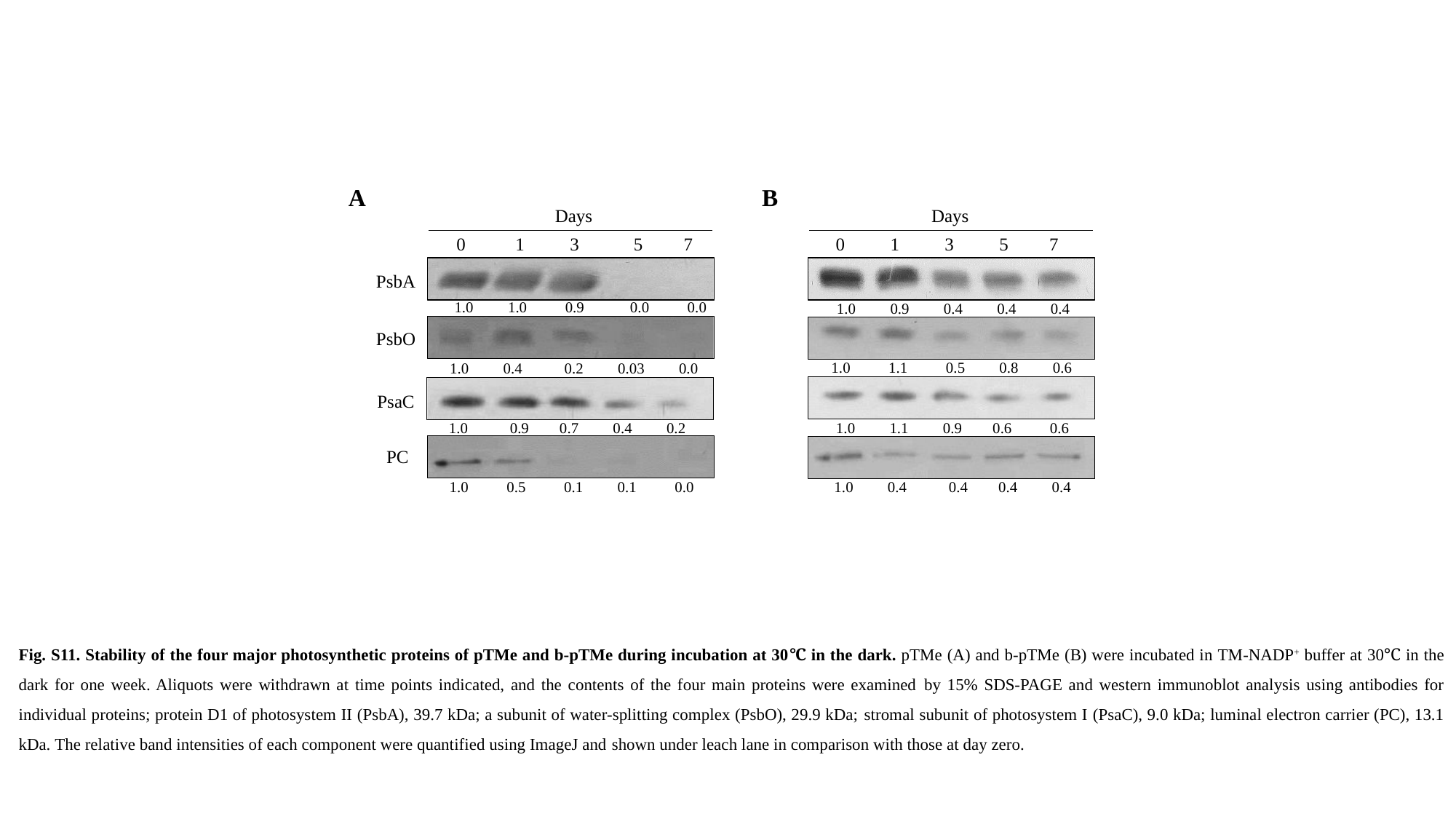

A
B
Days
Days
 0 1 3 5 7
 0 1 3 5 7
PsbA
1.0 1.0 0.9 0.0 0.0
1.0 0.9 0.4 0.4 0.4
PsbO
1.0 1.1 0.5 0.8 0.6
 1.0 0.4 0.2 0.03 0.0
PsaC
1.0 0.9 0.7 0.4 0.2
 1.0 1.1 0.9 0.6 0.6
PC
 1.0 0.4 0.4 0.4 0.4
1.0 0.5 0.1 0.1 0.0
Fig. S11. Stability of the four major photosynthetic proteins of pTMe and b-pTMe during incubation at 30℃ in the dark. pTMe (A) and b-pTMe (B) were incubated in TM-NADP+ buffer at 30℃ in the dark for one week. Aliquots were withdrawn at time points indicated, and the contents of the four main proteins were examined by 15% SDS-PAGE and western immunoblot analysis using antibodies for individual proteins; protein D1 of photosystem II (PsbA), 39.7 kDa; a subunit of water-splitting complex (PsbO), 29.9 kDa; stromal subunit of photosystem I (PsaC), 9.0 kDa; luminal electron carrier (PC), 13.1 kDa. The relative band intensities of each component were quantified using ImageJ and shown under leach lane in comparison with those at day zero.

## Slide 15
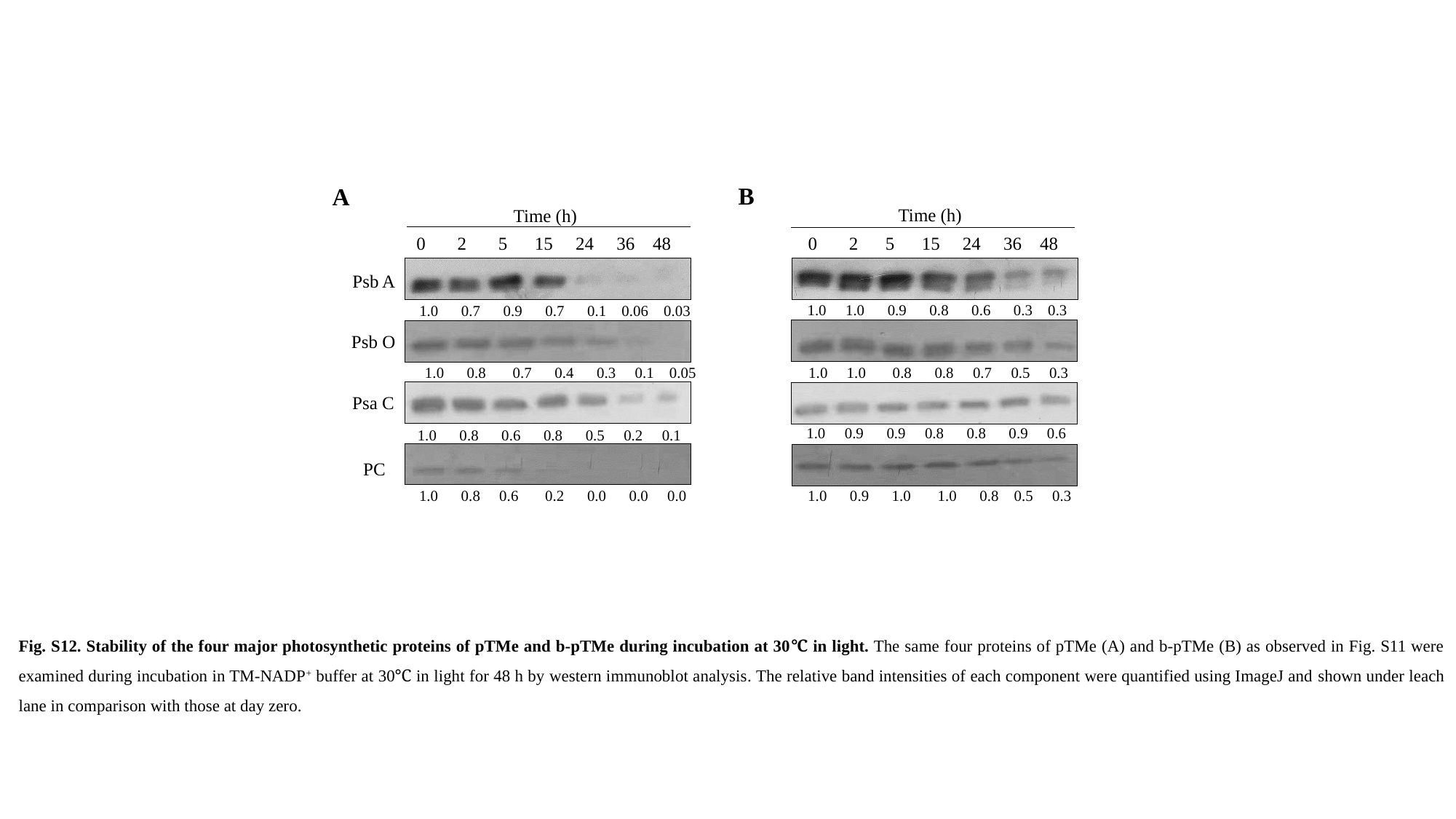

B
A
Time (h)
Time (h)
 0 2 5 15 24 36 48
 0 2 5 15 24 36 48
Psb A
1.0 1.0 0.9 0.8 0.6 0.3 0.3
1.0 0.7 0.9 0.7 0.1 0.06 0.03
Psb O
1.0 1.0 0.8 0.8 0.7 0.5 0.3
 1.0 0.8 0.7 0.4 0.3 0.1 0.05
Psa C
1.0 0.9 0.9 0.8 0.8 0.9 0.6
1.0 0.8 0.6 0.8 0.5 0.2 0.1
PC
 1.0 0.8 0.6 0.2 0.0 0.0 0.0
1.0 0.9 1.0 1.0 0.8 0.5 0.3
Fig. S12. Stability of the four major photosynthetic proteins of pTMe and b-pTMe during incubation at 30℃ in light. The same four proteins of pTMe (A) and b-pTMe (B) as observed in Fig. S11 were examined during incubation in TM-NADP+ buffer at 30℃ in light for 48 h by western immunoblot analysis. The relative band intensities of each component were quantified using ImageJ and shown under leach lane in comparison with those at day zero.

## Slide 16
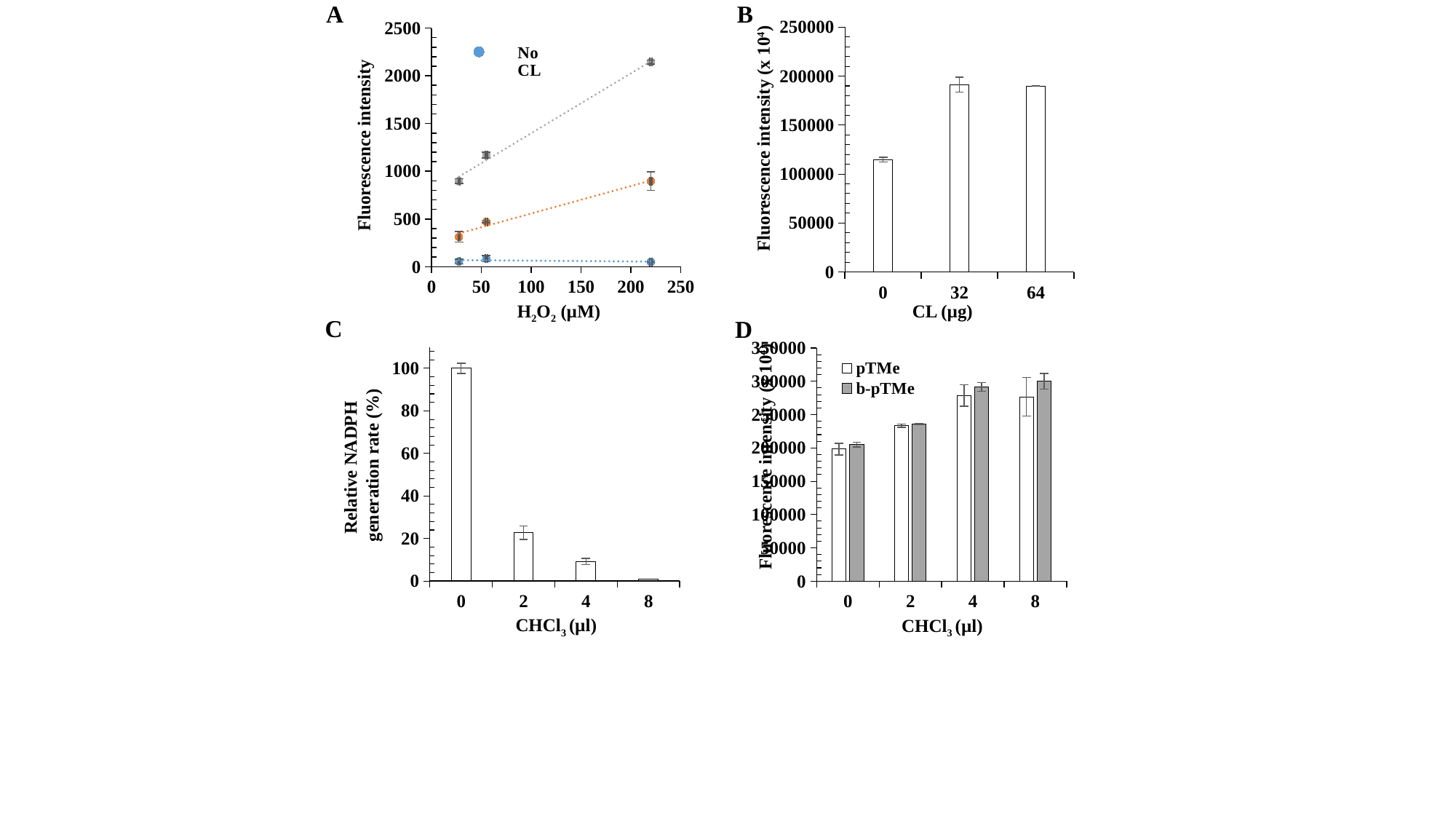

A
B
### Chart
| Category | |
|---|---|
| 0 | 114625.0 |
| 32 | 191244.0 |
| 64 | 189968.0 |
### Chart
| Category | No CL | 32 µg CL | 64 µg CL |
|---|---|---|---|Fluorescence intensity (x 104)
Fluorescence intensity
CL (µg)
H2O2 (µM)
C
D
### Chart
| Category | pTMe | b-pTMe |
|---|---|---|
| 0 | 198188.0 | 205088.0 |
| 2 | 233562.0 | 235472.0 |
| 4 | 278760.0 | 291528.0 |
| 8 | 276686.0 | 299940.0 |
### Chart
| Category | |
|---|---|
| 0 | 100.0 |
| 2 | 22.70114942528738 |
| 4 | 9.195402298850585 |
| 8 | 1.0 |Relative NADPH
generation rate (%)
Fluorescence intensity (x 104)
CHCl3 (µl)
CHCl3 (µl)

## Slide 17
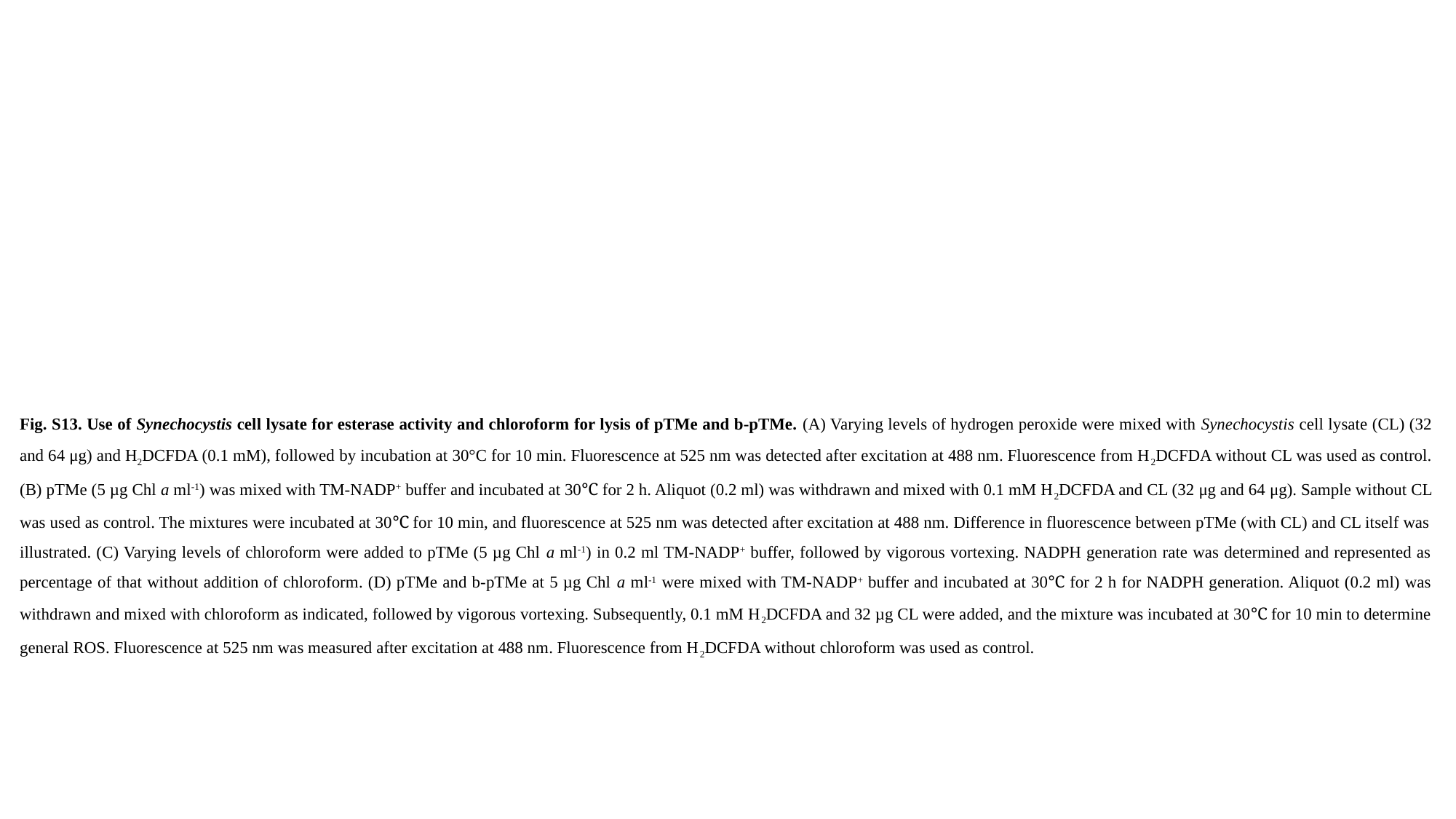

Fig. S13. Use of Synechocystis cell lysate for esterase activity and chloroform for lysis of pTMe and b-pTMe. (A) Varying levels of hydrogen peroxide were mixed with Synechocystis cell lysate (CL) (32 and 64 μg) and H2DCFDA (0.1 mM), followed by incubation at 30°C for 10 min. Fluorescence at 525 nm was detected after excitation at 488 nm. Fluorescence from H2DCFDA without CL was used as control. (B) pTMe (5 µg Chl a ml-1) was mixed with TM-NADP+ buffer and incubated at 30℃ for 2 h. Aliquot (0.2 ml) was withdrawn and mixed with 0.1 mM H2DCFDA and CL (32 μg and 64 μg). Sample without CL was used as control. The mixtures were incubated at 30℃ for 10 min, and fluorescence at 525 nm was detected after excitation at 488 nm. Difference in fluorescence between pTMe (with CL) and CL itself was illustrated. (C) Varying levels of chloroform were added to pTMe (5 µg Chl a ml-1) in 0.2 ml TM-NADP+ buffer, followed by vigorous vortexing. NADPH generation rate was determined and represented as percentage of that without addition of chloroform. (D) pTMe and b-pTMe at 5 µg Chl a ml-1 were mixed with TM-NADP+ buffer and incubated at 30℃ for 2 h for NADPH generation. Aliquot (0.2 ml) was withdrawn and mixed with chloroform as indicated, followed by vigorous vortexing. Subsequently, 0.1 mM H2DCFDA and 32 µg CL were added, and the mixture was incubated at 30℃ for 10 min to determine general ROS. Fluorescence at 525 nm was measured after excitation at 488 nm. Fluorescence from H2DCFDA without chloroform was used as control.

## Slide 18
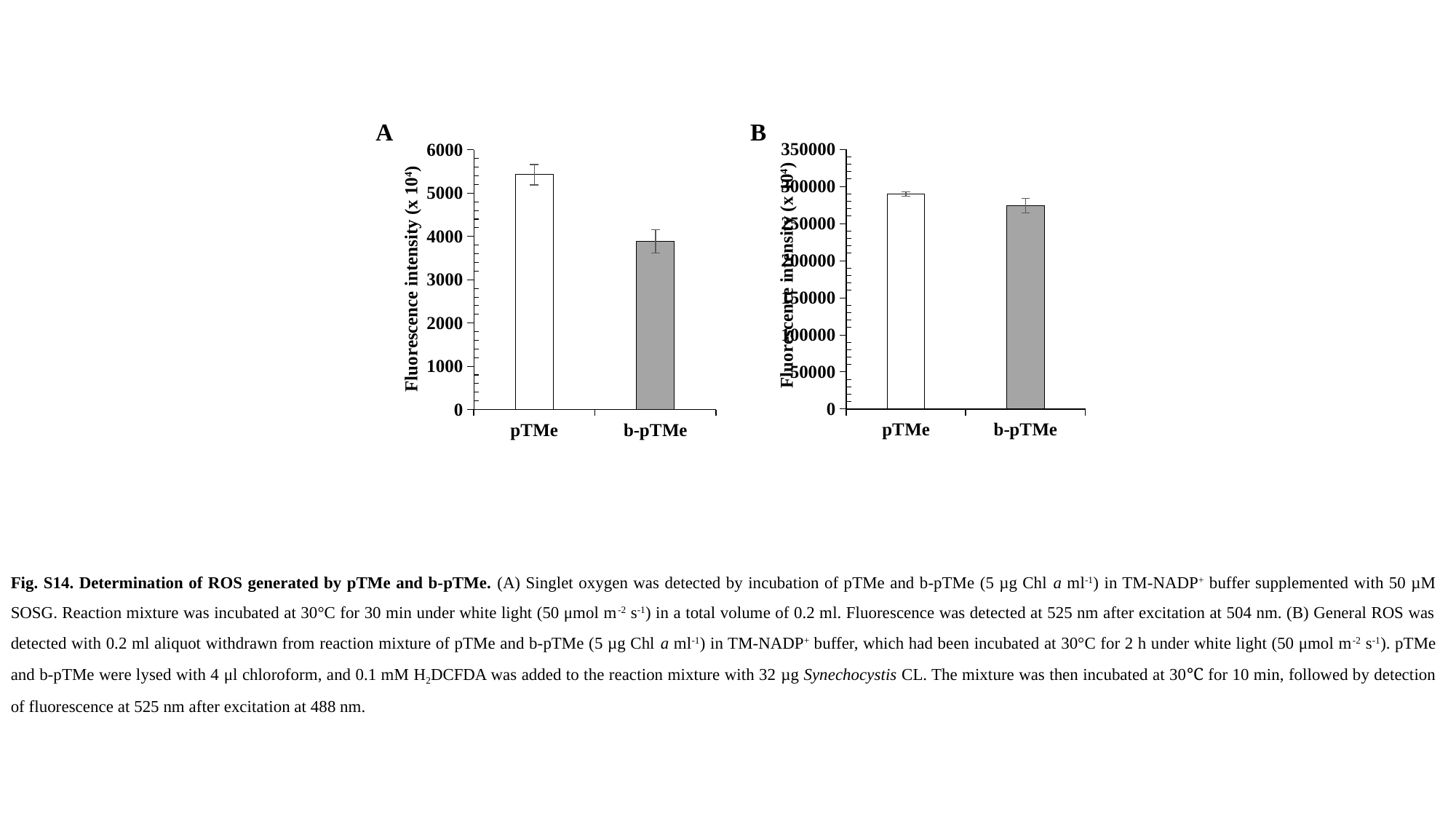

A
B
### Chart
| Category | |
|---|---|
| pTMe | 5426.5 |
| b-pTMe | 3889.5 |
### Chart
| Category | |
|---|---|
| pTMe | 289806.0 |
| b-pTMe | 274082.0 |Fluorescence intensity (x 104)
Fluorescence intensity (x 104)
Fig. S14. Determination of ROS generated by pTMe and b-pTMe. (A) Singlet oxygen was detected by incubation of pTMe and b-pTMe (5 µg Chl a ml-1) in TM-NADP+ buffer supplemented with 50 µM SOSG. Reaction mixture was incubated at 30°C for 30 min under white light (50 μmol m-2 s-1) in a total volume of 0.2 ml. Fluorescence was detected at 525 nm after excitation at 504 nm. (B) General ROS was detected with 0.2 ml aliquot withdrawn from reaction mixture of pTMe and b-pTMe (5 µg Chl a ml-1) in TM-NADP+ buffer, which had been incubated at 30°C for 2 h under white light (50 μmol m-2 s-1). pTMe and b-pTMe were lysed with 4 μl chloroform, and 0.1 mM H2DCFDA was added to the reaction mixture with 32 µg Synechocystis CL. The mixture was then incubated at 30℃ for 10 min, followed by detection of fluorescence at 525 nm after excitation at 488 nm.

## Slide 19
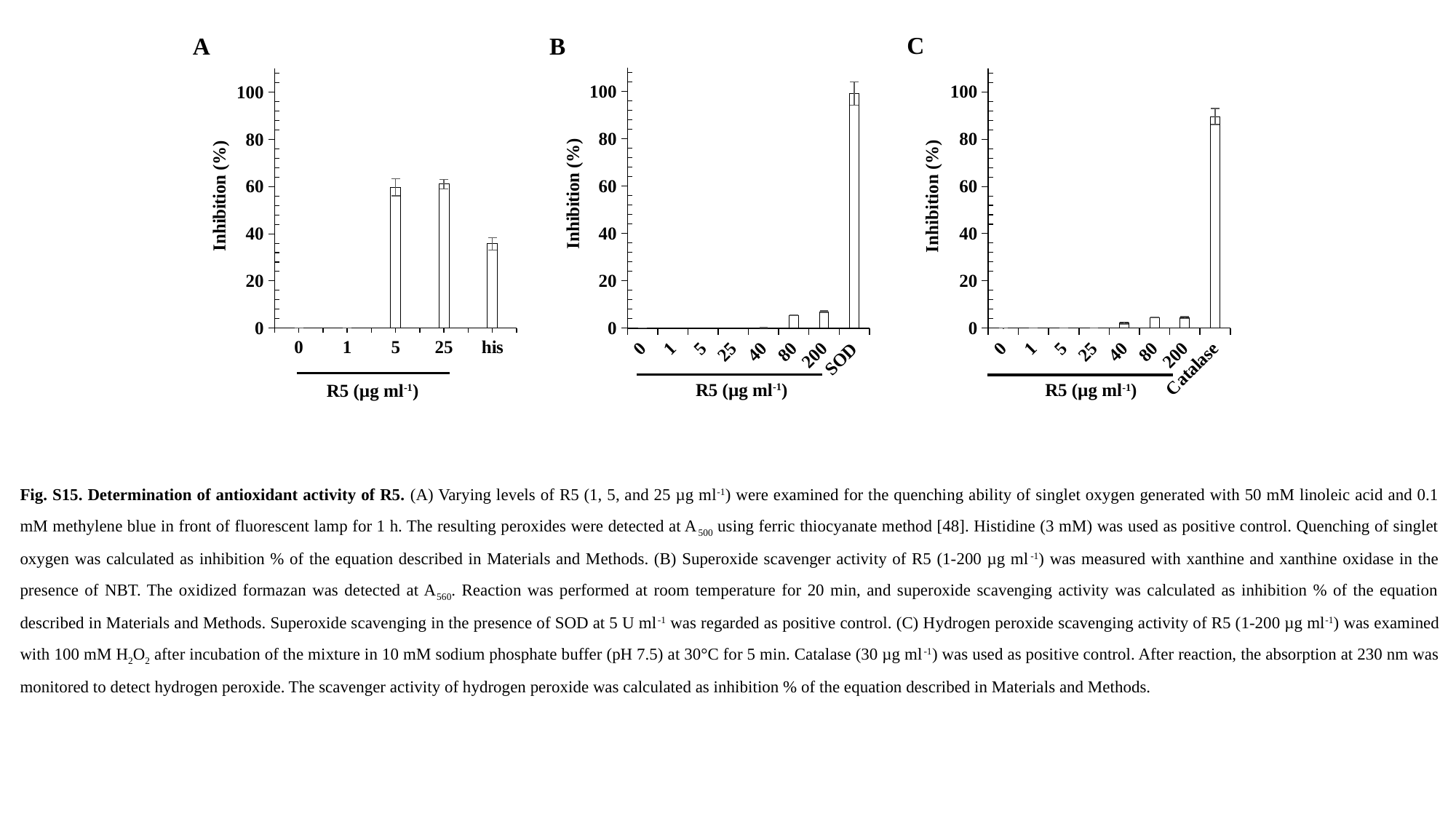

C
A
B
### Chart
| Category | |
|---|---|
| 0 | 0.01 |
| 1 | 0.01 |
| 5 | 59.74025974025983 |
| 25 | 61.038961038961 |
| his | 35.71428571428569 |
### Chart
| Category | |
|---|---|
| 0 | 0.01 |
| 1 | 0.1 |
| 5 | 0.1 |
| 25 | 0.1 |
| 40 | 0.217864923747277 |
| 80 | 5.446623093681924 |
| 200 | 6.971677559912862 |
| SOD | 99.12854030501087 |
### Chart
| Category | |
|---|---|
| 0 | 0.0367 |
| 1 | 0.0587 |
| 5 | 0.0469 |
| 25 | 0.109 |
| 40 | 2.104499274310605 |
| 80 | 4.499274310595073 |
| 200 | 4.426705370101603 |
| Catalase | 89.55007256894048 |Inhibition (%)
R5 (µg ml-1)
R5 (µg ml-1)
R5 (µg ml-1)
Fig. S15. Determination of antioxidant activity of R5. (A) Varying levels of R5 (1, 5, and 25 µg ml-1) were examined for the quenching ability of singlet oxygen generated with 50 mM linoleic acid and 0.1 mM methylene blue in front of fluorescent lamp for 1 h. The resulting peroxides were detected at A500 using ferric thiocyanate method [48]. Histidine (3 mM) was used as positive control. Quenching of singlet oxygen was calculated as inhibition % of the equation described in Materials and Methods. (B) Superoxide scavenger activity of R5 (1-200 µg ml-1) was measured with xanthine and xanthine oxidase in the presence of NBT. The oxidized formazan was detected at A560. Reaction was performed at room temperature for 20 min, and superoxide scavenging activity was calculated as inhibition % of the equation described in Materials and Methods. Superoxide scavenging in the presence of SOD at 5 U ml-1 was regarded as positive control. (C) Hydrogen peroxide scavenging activity of R5 (1-200 µg ml-1) was examined with 100 mM H2O2 after incubation of the mixture in 10 mM sodium phosphate buffer (pH 7.5) at 30°C for 5 min. Catalase (30 µg ml-1) was used as positive control. After reaction, the absorption at 230 nm was monitored to detect hydrogen peroxide. The scavenger activity of hydrogen peroxide was calculated as inhibition % of the equation described in Materials and Methods.

## Slide 20
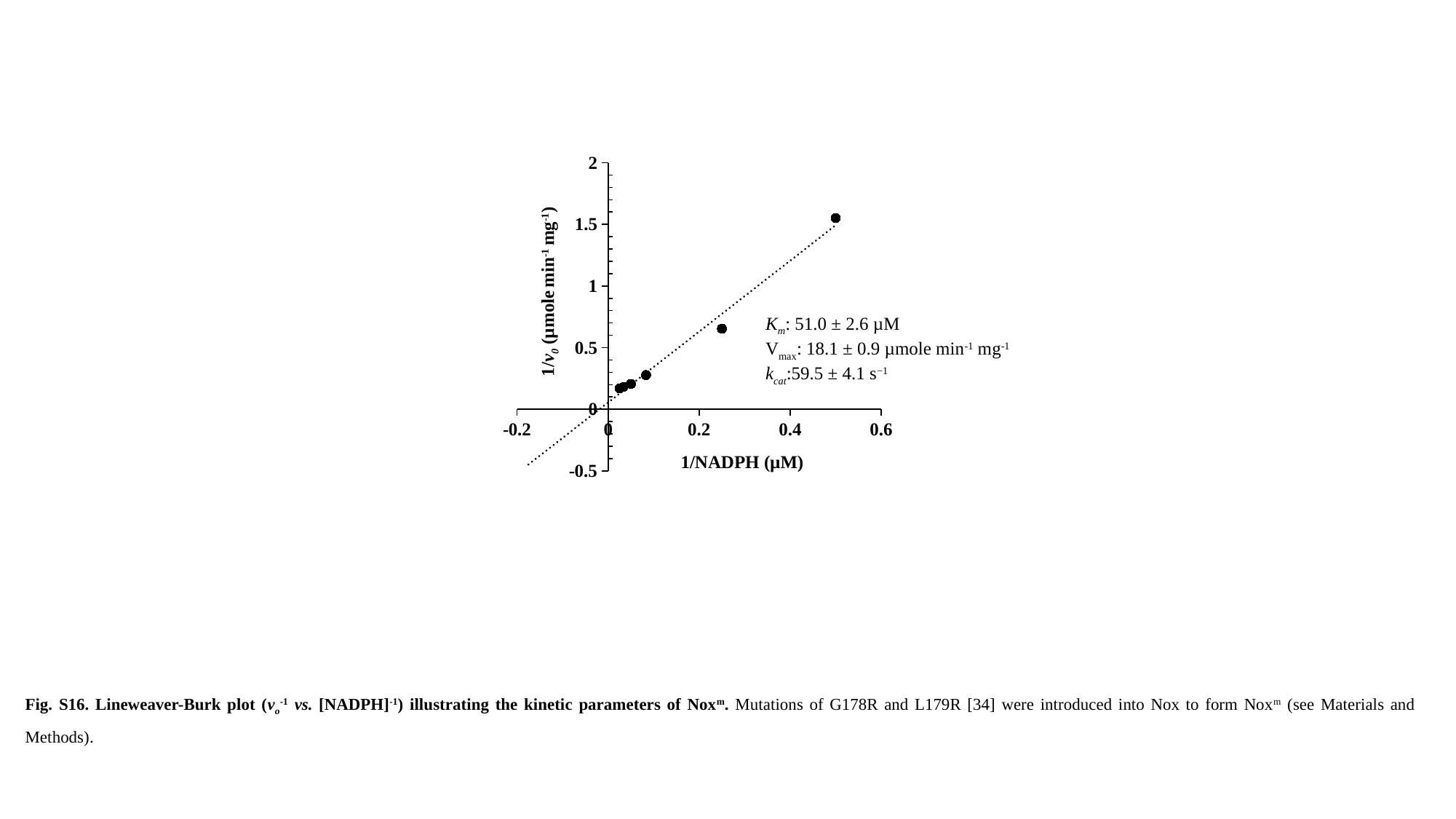

### Chart
| Category | |
|---|---|1/v0 (µmole min-1 mg-1)
Km: 51.0 ± 2.6 µM
Vmax: 18.1 ± 0.9 µmole min-1 mg-1
kcat:59.5 ± 4.1 s−1
1/NADPH (µM)
Fig. S16. Lineweaver-Burk plot (vo-1 vs. [NADPH]-1) illustrating the kinetic parameters of Noxm. Mutations of G178R and L179R [34] were introduced into Nox to form Noxm (see Materials and Methods).

## Slide 21
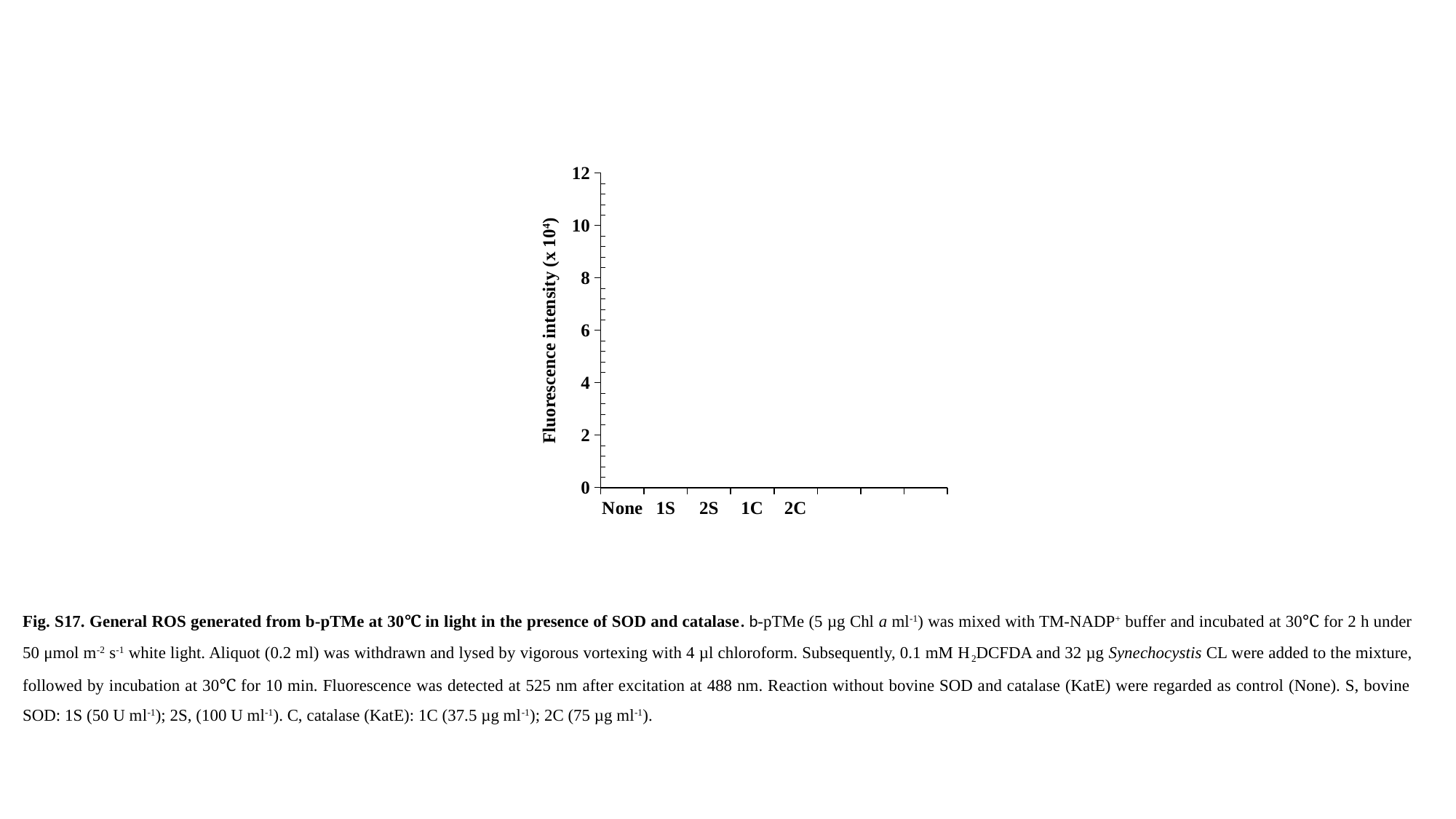

### Chart
| Category | |
|---|---|
| None | 245887.56756756754 |
| 1S | 247406.04395604396 |
| 2S | 246932.5 |
| 1C | 104518.24324324324 |
| 2C | 101767.76315789475 |Fluorescence intensity (x 104)
Fig. S17. General ROS generated from b-pTMe at 30℃ in light in the presence of SOD and catalase. b-pTMe (5 µg Chl a ml-1) was mixed with TM-NADP+ buffer and incubated at 30℃ for 2 h under 50 μmol m-2 s-1 white light. Aliquot (0.2 ml) was withdrawn and lysed by vigorous vortexing with 4 µl chloroform. Subsequently, 0.1 mM H2DCFDA and 32 µg Synechocystis CL were added to the mixture, followed by incubation at 30℃ for 10 min. Fluorescence was detected at 525 nm after excitation at 488 nm. Reaction without bovine SOD and catalase (KatE) were regarded as control (None). S, bovine SOD: 1S (50 U ml-1); 2S, (100 U ml-1). C, catalase (KatE): 1C (37.5 µg ml-1); 2C (75 µg ml-1).
